# The Plausibility of Sampling as an Algorithmic Theory of Sentence Processing

**DOI:** 10.1162/opmi_a_00086

**Published:** 2023-07-21

**Authors:** Jacob Louis Hoover, Morgan Sonderegger, Steven T. Piantadosi, Timothy J. O’Donnell

**Affiliations:** McGill University, Montréal, Canada; Mila Québec AI Institute, Montréal, Canada; University of California Berkeley, Berkeley, CA, USA; Canada CIFAR AI Chair, Mila

**Keywords:** sentence processing, parsing algorithms, sampling, surprisal

## Abstract

Words that are more surprising given context take longer to process. However, no incremental parsing algorithm has been shown to directly predict this phenomenon. In this work, we focus on a class of algorithms whose runtime does naturally scale in surprisal—those that involve repeatedly sampling from the prior. Our first contribution is to show that simple examples of such algorithms predict runtime to increase superlinearly with surprisal, and also predict variance in runtime to increase. These two predictions stand in contrast with literature on surprisal theory (Hale, 2001; Levy, 2008a) which assumes that the expected processing cost increases linearly with surprisal, and makes no prediction about variance. In the second part of this paper, we conduct an empirical study of the relationship between surprisal and reading time, using a collection of modern language models to estimate surprisal. We find that with better language models, reading time increases superlinearly in surprisal, and also that variance increases. These results are consistent with the predictions of sampling-based algorithms.

## INTRODUCTION

One of the fundamental problems of computational psycholinguistics, going back to the earliest days of the field, is to provide an algorithmic theory of human sentence processing (see e.g., Collins & Roark, 2004; Dotlačil, 2021; Frazier & Fodor, 1978; Lewis & Vasishth, 2005; Marcus, 1978; Miller & Chomsky, 1963; Rasmussen & Schuler, 2018; Roark, 2001; Stolcke, 1995; Vasishth & Engelmann, 2021; Yngve, 1960). Such an algorithmic theory must satisfy a number of important empirical constraints. Amongst these are that the human processor is *incremental* and *predictive*—people process sentences eagerly, assigning as much meaning as possible as early as possible, and predicting likely continuations based on the current context (Eberhard et al., 1995; Frazier, 1987; Marslen-Wilson, 1973, 1975; Tanenhaus et al., 1995). Moreover, the effort needed to integrate each subsequent word (or smaller unit) depends on how predictable it is, in context, often quantified as *surprisal* (negative log probability given context; Hale, 2001; Levy, 2008a). The more surprising a word is, the more time it takes to integrate (e.g., Balota et al., 1985; Brothers & Kuperberg, 2021; Ehrlich & Rayner, 1981; McDonald & Shillcock, 2003a, 2003b; Meister et al., 2021; Wilcox et al., 2020).

However, despite the widespread recognition of these empirical facts, and the large number of studies looking at surprisal as an empirical predictor of incremental processing time (e.g., Demberg & Keller, 2008; Goodkind & Bicknell, 2018, 2021; Hofmann et al., 2022; Meister et al., 2021; Smith & Levy, 2008a, 2013; Wilcox et al., 2020), to our knowledge no sentence processing algorithm has been proposed for which incremental runtime intrinsically increases as a function of surprisal.

In Sampling Algorithms for Sentence Processing, we review the kinds of algorithms that could possibly possess the desired properties, identifying and focusing on a class of approaches for which the desired relationship with surprisal is very natural—sampling based algorithms. The first contribution of this paper is to show that under some reasonable assumptions, sampling-based algorithms predict processing time to be a monotonic increasing function of surprisal. In particular, these algorithms predict runtime to increase as a superlinear function of surprisal. We also show that these algorithms make a novel prediction about processing times—under sampling based algorithms, we also expect variance to increase with surprisal.

However, as we discuss in Surprisal Theory, these two predictions are inconsistent with the assumptions made by the majority of published work in surprisal theory. In particular, empirical studies in this area have often assumed that the relationship between surprisal and processing time is linear (Demberg & Keller, 2008; Fernandez Monsalve et al., 2012; Frank et al., 2013), or at least that variance is constant (Goodkind & Bicknell, 2018; Meister et al., 2021; Smith & Levy, 2008a, 2013; Wilcox et al., 2020). We review the status of the widespread assumptions of linearity and constant variance, identifying both theoretical and empirical reasons to question these properties.

We then present a new targeted study of the empirical relationship between surprisal and reading time (in Empirical Study). We obtain surprisal estimates from a variety of pre-trained language models (LMs), including GPT-3 (Brown et al., 2020) and then use generalized additive models (Wood et al., 2016) to examine the shape of the linking function between surprisal and reading time. We control for possibly nonlinear by-subject random effects, and also fit the relationship between surprisal and variance in reading time. We find evidence that the overall shape of the linking function is in fact superlinear, especially for surprisals estimated by the most accurate LMs. We additionally find that variance in reading time increases with surprisal. Both these results are at odds with the assumptions typically made in surprisal theory, but they are consistent with the predictions of sampling-based algorithms for processing.

We situate our results in the context of earlier literature, speculating that our ability to detect this superlinear relationship rests on several ways our empirical study improves upon previous work. Namely, we use higher quality LMs to estimate surprisal, and fit statistical models designed to assess the possibly nonlinear relationship, controlling for individual differences. In the discussion, we also revisit previous proposals which are related to the analyses we give of sampling algorithms. Based on our theoretical and empirical results, we propose that sampling-based mechanisms form a promising yet under-explored family of algorithms for the modelling of human sentence processing.

## SAMPLING ALGORITHMS FOR SENTENCE PROCESSING

It is well documented that for humans, words that are less expected are harder to process—for example, during reading, people spend more time looking at words which are less predictable given context (e.g., Balota et al., 1985; Brothers & Kuperberg, 2021; Ehrlich & Rayner, 1981; Goodkind & Bicknell, 2018; Hofmann et al., 2022; McDonald & Shillcock, 2003a, 2003b; Meister et al., 2021; Smith & Levy, 2013; Wilcox et al., 2020). We may write this general relationship as:

$$\text{Time}\left(w_{n}\right)\approx f\left(I\left(w_{n}\right)\right)$$ (1)

where the linking function *f* is some monotonically increasing function, and

$$I\left(w_{n}\right)≔-\log\;p\left(w_{n}|w_{1:n-1}\right)$$ (2)

is the *surprisal* of word *w*_*n*_. Thus, we seek an algorithmic model of sentence processing where the computational cost to perform each incremental update depends on the surprisal of the input at that step.

To clarify what is at stake, it is useful to consider the incremental sentence processing problem in more detail. Sentence processing can be viewed as a sequence of posterior inference problems: The comprehender updates their beliefs about the intended meaning, parse, or other latent structure as they successively observe linguistic input items (e.g., words, morphemes, or smaller units). Formally, we can define a probabilistic incremental parser as a map which, at each step, takes the sequence of linguistic inputs seen so far to a posterior distribution: *w*_1:*n*_ ↦ *p*(*z*|*w*_1:*n*_), where *z* ranges over meanings (or parses, etc.). Consider one step of this process, assuming that the comprehender has a representation of the exact posterior distribution given *w*_1:*n*−1_, then encounters the next word *w*_*n*_. The job of this comprehender is to update their beliefs about meanings in light of the evidence, to obtain a new posterior:

$$p\left(z|w_{1:n}\right)=\frac{p\left(w_{n}|z\right)p\left(z|w_{1:n-1}\right)}{\sum_{z}p\left(w_{n}|z\right)p\left(z|w_{1:n-1}\right)}$$ (3)

Note that the denominator here is ∑_*z*_ *p*(*w*_*n*_|*z*)*p*(*z*|*w*_1:*n*−1_) = *p*(*w*_*n*_|*w*_1:*n*−1_), the marginal probability of the word given the preceding context—the negative logarithm of this quantity is the surprisal. This denominator represents the proportion of the prior meaning space that remains after posterior update. When it is small (and thus surprisal is high), this means that very little of the prior meaning space *p*(*z*|*w*_1:*n*−1_) was consistent with the new word, when it is large (and thus surprisal is low), this means that much of the prior meaning space was consistent with the new word.

### Algorithms That Do Not Scale in Surprisal

In the literature studying surprisal and processing cost, it has been common to use enumerative algorithms, such as Stolcke’s probabilistic variant of Earley’s chart-based algorithm (Earley, 1970; Stolcke, 1995) to estimate surprisal values (e.g., Boston et al., 2008; Levy, 2008a). Without further assumptions such as probability-based pruning (see below), such enumerative algorithms do not use the probability of chart items in deciding how much work to do, and thus do not scale in surprisal. The number of steps such an algorithm takes to integrate the next word into the chart can depend on the size and specification of a probabilistic grammar, but cannot depend on the probability of the word. This is also true of the many probabilistic or non-probabilistic bottom-up, top-down, or left corner parsing algorithms which have been studied over the years as models of sentence processing (Abney & Johnson, 1991; Berwick & Weinberg, 1982; Earley, 1970; Graf et al., 2017; Marcus, 1978; Nivre, 2008; Roark, 2001; Rosenkrantz & Lewis, 1970; Stabler, 2013), and likewise for RNN- or Transformer-based parsing models (e.g., Costa, 2003; Hu et al., 2021, 2022; Jin & Schuler, 2020; Yang & Deng, 2020).

Other parsing algorithms have properties which result in some correlation between surprisal and processing cost, without predicting the relationship directly. For instance, amortized parsing techniques that make use of *chunked* (Newell & Paul, 1981) parser moves or grammar fragments (as examined in, e.g., Hale, 2014; Luong et al., 2015), can predict broadly that common sequences of actions lead to lower surprisal. However, these accounts do not predict any direct link between individual word probability and the amount of computational work done by the processor. A similar argument can be made for theories which describe processing difficulty primarily in terms of distance-based measures such as dependency locality theory (DLT; Gibson, 1998, 2000), where certain common words may tend to have shorter dependencies, but the surprisal of a word is not intrinsically related to its integration cost.

A final class of models to consider includes causal language models, which do not produce any observable representations of the meaning of their input, but rather simply predict the next word given some prefix (Brown et al., 2020; Dai et al., 2019; Hochreiter & Schmidhuber, 1997; Radford et al., 2018, 2019). The amount of work required by these algorithms may scale in quantities such as the length of the input or the size of the vocabulary, or other functions of the architecture of the model, but never directly as a function of the probability of the next word.

### Algorithms That Do Scale in Surprisal

As outlined above, highly probable words will necessarily tend to be associated with more likely meanings (parses) given the preceding words, while the least likely words will tend to be less compatible with these meanings. This suggests a natural way to relate processing algorithms’ computational cost to the surprisal of the next word: When doing the posterior update, give priority to those meanings which are highly likely in the prior *p*(*z*|*w*_1:*n*−1_). Since a word *w*_*n*_ with low surprisal will tend to be associated with highly probable prior meanings, privileging meanings in such a way will lead to algorithms with the desired dependence on surprisal.

In this work we focus on a broad class of algorithms that privilege high prior probability meanings: those that *sample* candidate meanings from the prior distribution *p*(*z*|*w*_1:*n*−1_).^1^ Another closely related class of algorithms with this property are those which perform a deterministic search over the space of meanings, in order of decreasing prior probability. Such an algorithm will naturally tend to take longer when confronted with an input word that has higher surprisal (see discussion in Deterministic Search Algorithms).

In what follows, we will consider two simple procedures for sampling from the prior and discuss their consequences for theories of incremental sentence processing.

### Two Simple Sampling Algorithms

In the analyses that follow, we consider the problem of integrating a single word *w*_*n*_ assuming that the comprehender has an exact representation of the true prior: *p*(*z*|*w*_1:*n*−1_). Note that the probability that a random sample from the true prior will be consistent with observed word *w*_*n*_ is given by ∑_*z*_ *p*(*w*_*n*_|*z*)*p*(*z*|*w*_1:*n*−1_) = *p*(*w*_*n*_|*w*_1:*n*−1_). Thus, without loss of generality, we simplify the problem to analyzing the expected number of samples needed to exactly match *w*_*n*_. Note, assuming an exact prior representation is highly conservative, since, in general, sampling-based algorithms for incremental processing will have to be approximate (e.g., using Markov chain or sequential Monte Carlo techniques) and so will accumulate errors. A similar observation can be made about modified versions of these algorithms which sample until some constant number of successes are achieved (rather than stopping at the first success). The runtime analyses we do here will thus provide a lower bound on runtime for the more general class of algorithms.

#### Simple guessing algorithm.

Define the simple guessing algorithm^2^ as follows: To get an exact sample from posterior *p*(·|*w*_1:*n*_), given prior *p*(·|*w*_1:*n*−1_), and observed next word *w*_*n*_, repeatedly sample hypotheses (meanings) from the prior until getting one which explains the observed next word.^3^

The number of samples needed in this scheme, *M*, is geometrically distributed *M* ∼ *Geom*(*p*), where parameter *p* = *p*(*w*_*n*_|*w*_1:*n*−1_) is the probability of success. This random variable has expected value 1/*p* and variance (1 − *p*)/*p*^2^. Expressed as a function of surprisal, the expected value and variance are

$$𝔼\left[M\right]=\frac{1}{p}=e^{I\left(w_{n}\right)}$$ (4)

$$\text{Var}\left[M\right]=\frac{1-p}{p^{2}}=e^{2I\left(w_{n}\right)}-e^{I\left(w_{n}\right)}$$ (5)

So, the expected runtime of this sampling scheme (eq. 4) increases monotonically—in fact, exponentially—in surprisal. Likewise, the variance in runtime (eq. 5) also increases monotonically and superlinearly as a function of surprisal (to see this, note that all its derivatives are everywhere positive).

#### Guessing without replacement algorithm.

In the simple guessing algorithm above, a meaning may be repeatedly sampled from the prior, despite not explaining the observation. So, we will also consider a more efficient version of the above scheme where sampling is carried out *without replacement* to avoid re-sampling meanings that have already been eliminated.

Define the simple guessing algorithm without replacement as follows: Let the meanings which do not explain the observation be indexed 1, …, *K*, with weights ${\left\{u_{i}\right\}}_{i=1}^{K}$. Consider one additional item, the target, assigned index 0, with weight, *u*_0_, proportional to the total probability mass of the meanings which do explain the observation. At each step of the algorithm an item is sampled from the set {0, …, *K*} with probabilities proportional to the weights of the items not yet drawn. The algorithm halts when the target item (0) is drawn.

Define binary random variables ${\left\{X_{i}\right\}}_{1}^{K}$. where *X*_*i*_ = 1 if item *k* is drawn before the target, else *X*_*i*_ = 0. Let random variable *N* be the number of guesses without replacement up to and including when the target is drawn. Then the runtime *N* = 1 + $\sum_{i=1}^{K}$ *X*_*i*_.

To derive runtime mean and variance for this algorithm, the following proposition will be useful.

**Proposition 1.** *In a guessing algorithm (with or without replacement) the probability of drawing item i before item j is* Pr(*i* ≺ *j*) = $\frac{u_{i}}{u_{i}+u_{j}}$.^4^

*Proof.* Consider a modification of the guessing-without-replacement scheme in which items *i* and *j* have been removed from the set and a new item *i* ∨ *j*. is inserted instead, with weight *u*_*i*_ + *u*_*j*_. If this item is drawn, then we say *i* is drawn with probability Pr(*i*|*i* ∨ *j*) = $\frac{u_{i}}{u_{i}+u_{j}}$, else *j* is drawn. The runtime of this scheme is identical to that of guessing without replacement. Let *S*_*K*−1_ be the set of permutations of ({0, …, *K*} \ {*i*, *j*}) ∪ {{*i* ∨ *j*}}. First note that for any permutation *σ* ∈ *S*_*K*−1_, the conditional probability Pr(*i* ≺ *j*|*σ*) = Pr(*i*|*i* ∨ *j*). So Pr(*i* ≺ *j*) = ∑_*σ*_ Pr(*i* ≺ *j*|*σ*) Pr(*σ*) = Pr(*i*|*i* ∨ *j*) = $\frac{u_{i}}{u_{i}+u_{j}}$.

So, with 𝔼[*X*_*i*_] = Pr(*i* ≺ 0) = $\frac{u_{i}}{u_{i}+u_{0}}$, we have that the expected runtime (number of draws), is

$$\begin{array}{l} 𝔼\left[N\right]=𝔼\left[1+\sum_{i}X_{i}\right]=1+\sum_{i}𝔼\left[X_{i}\right]\\ \;=1+\sum_{i}\frac{u_{i}}{u_{i}+u_{0}} \end{array}$$ (6)

and the variance in number of draws is

$$\begin{array}{l} \text{Var}[N]=\sum_{i}\left[𝔼[X_{i}]-{\left(𝔼[X_{i}]\right)}^{2}\right]+\underset{i\neq j}{\sum }\left[𝔼[X_{i}X_{j}]-𝔼[X_{i}]𝔼[X_{j}]\right]\\ \;=\underset{i}{\sum }\left[\frac{u_{i}}{u_{i0}}-{\left(\frac{u_{i}}{u_{i0}}\right)}^{2}\right]+\underset{i\neq j}{\sum }\left[\frac{u_{i}}{u_{ij0}}\frac{u_{j}}{u_{ij0}}+\frac{u_{j}}{u_{ij0}}\frac{u_{i}}{u_{i0}}-\frac{u_{i}}{u_{i0}}\frac{u_{j}}{u_{j0}}\right] \end{array}$$ (7)

using notation *u*_*ab*_ := *u*_*a*_ + *u*_*b*_ and *u*_*abc*_ := *u*_*a*_ + *u*_*b*_ + *u*_*c*_. See Appendix A for a derivation.

An important property to note here is that the individual weights of all items ${\left\{u_{i}\right\}}_{i=0}^{K}$ appear in the general expressions for mean runtime (eq. 6) and variance in runtime (eq. 7). This means that both mean and variance in runtime depend on how the weights are distributed across all the items—not just the probability of success, as was the case in the simple guessing (with replacement) algorithm. Obtaining a concrete prediction for how the runtime scales as a function of surprisal requires making some assumption about the distribution from which we are sampling.

**Figure 1.**
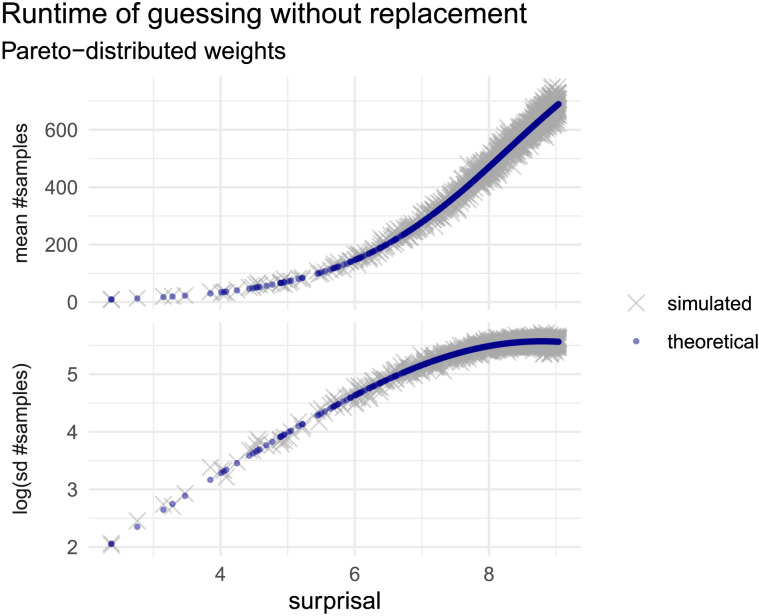
**Relationship between surprisal (negative log probability) and guessing-without-replacement runtime for a set of 1000 weights sampled from a *Pareto*(1, 1) distribution.** Blue points show theoretical values for mean (top) and variance (bottom, transformed as log standard deviation). Grey crosses give average values in simulating 500 runs of the algorithm for each surprisal value.

We will assume the item probabilities are heavy-tailed—specifically, that they are power-law distributed (a property ubiquitous in language, and word frequency distributions in particular; see Piantadosi, 2014). Figure 1 shows the empirical mean and variance of guessing-without-replacement runtime (number of samples until success) plotted against the surprisal of the target, for *K* = 1000 weights sampled from the power-law distribution *Pareto*(1, 1), and normalized. Each of the discrete values on the horizontal axis corresponds to the negative log probability of one item in the set. The mean runtime to sample that item as the target is plotted in the top panel, and variance in the bottom panel. Blue points mark the theoretical values according to mean and variance derived in eqs. 6 and 7, and grey crosses indicate simulated values (estimated by simulating 500 runs of the algorithm for each item as the target).

We observe that the runtime of guessing-without-replacement increases as a superlinear function of surprisal, as is the case for the simple guessing algorithm with replacement. We also see that variance increases over most of the range of surprisal values, plateauing at the highest values of surprisal. Broadly, with respect to variance, we can say simply that it increases with surprisal, for both the with- and without-replacement algorithms.

## SURPRISAL THEORY

The relationship between surprisal and human processing time has received attention in a large number of studies (Bicknell & Levy, 2010, 2012; Boston et al., 2008; Brothers & Kuperberg, 2021; Demberg & Keller, 2008; Fernandez Monsalve et al., 2012; Frank, 2009; Frank et al., 2013; Futrell, 2017; Futrell et al., 2020; Goodkind & Bicknell, 2018, 2021; Hale, 2001; Hofmann et al., 2017, 2022; Jin & Schuler, 2020; Jurafsky, 1996; Levy, 2005, 2008a, 2008b, 2011, 2013, 2018; Lowder et al., 2018; McDonald & Shillcock, 2003a, 2003b; J. Mitchell et al., 2010; Narayanan & Jurafsky, 2001, 2004; Rasmussen & Schuler, 2018; Reichle et al., 2003; Roark et al., 2009; Smith & Levy, 2008a, 2008b, 2013; van Schijndel & Linzen, 2021; Wilcox et al., 2020). We will refer to literature focusing on this relationship as work on *surprisal theory*. The question of the shape of the function linking surprisal and processing time goes back to early work in the area (Hale, 2001; Levy, 2005; Narayanan & Jurafsky, 2004). The majority of work, however, has either assumed or explicitly argued for a linear linking function, that is,

$$\text{Time}\left(w_{n}\right)=\alpha +\beta I\left(w_{n}\right)$$ (8)

for some constants *α* and *β*. This stands in contrast with the superlinear linking function predicted by sampling-based mechanisms, described above. A linear relationship has been motivated both empirically and on the basis of theoretical arguments. Nevertheless, as we review below, there are reasons to question the assumption of linearity, including relatively recent studies that provide evidence of a superlinear linking function as well as earlier theoretical models that have assumed or argued for superlinearity (see Superlinearity in Surprisal Theory). Furthermore, as we note below, nearly all previous work has assumed the relationship between surprisal and variance in processing time to be constant.

### Empirical Studies in Surprisal Theory

Determining the correct functional relationship between surprisal and processing time is a long-standing problem in the field. A large number of studies have simply assumed a linear relationship, explicitly—or implicitly, by the use of linear statistical models for their analysis (e.g., Demberg & Keller, 2008; Frank, 2009; Fernandez Monsalve et al., 2012; Frank et al., 2013; Hao et al., 2020; Kuribayashi et al., 2022; Lowder et al., 2018; D. C. Mitchell, 1984; Reichle et al., 2003; van Schijndel & Linzen, 2021).^5^ A smaller number of papers, beginning with (Smith & Levy, 2008a, 2013), have investigated the shape of the linking function directly, using generalized additive models (GAMs; Wood, 2004, 2017), a family of statistical models which allows the fitting of arbitrary nonlinear relationships (Goodkind & Bicknell, 2018; Hofmann et al., 2022; Smith & Levy, 2008a, 2013; Wilcox et al., 2020). For the most part, these studies have found support for the assumption of linearity. However, there are a number of methodological reasons to revisit these results.

First, none of these previous studies has attempted a quantitative measure of superlinearity, relying instead on visual impression of the fitted curves. For instance, Goodkind and Bicknell (2018) and Wilcox et al. (2020) used nonlinear models to qualitatively confirm that the relationship looked linear before using linear models for interpretation.

Second, there is considerable variability between individuals in reading times and other psychometric measures of language processing (see Farmer et al., 2012). While GAMs allow the fitting an overall effect while controlling for arbitrary nonlinear by-subject effects, previous studies have either not controlled for such effects, (Hofmann et al., 2022; Smith & Levy, 2013; Wilcox et al., 2020),^6^ or assumed they were just constant offsets (Goodkind & Bicknell, 2018).

Third, all previous studies make strong assumptions about variance. Nearly all earlier studies have assumed that variance is constant, and normally distributed. A noteworthy exception is (Hofmann et al., 2022), who used a Gamma-distributed response distribution, which instead encodes the assumption that variance increases proportional to the square of the predicted reading time value. Smith and Levy (2013) also mention that their results are robust to switching to an assumption of Gamma-distributed response, though they do not report results of this modelling choice. As far as we are aware, no previous study has explored the form of the effect surprisal has on variance in processing time.

Fourth and finally, many of the earlier studies that examined the shape of the linking function directly using GAMs, notably including Smith and Levy (2008a, 2013), used surprisal estimates from trigram language models, which are far from current state-of-the-art. Modern pre-trained LMs allow unprecedentedly accurate prediction of words in context (see e.g., Brown et al., 2020; Floridi & Chiriatti, 2020). While questions remain about the similarity between even the best modern LM’s predictions and those of humans, numerous studies in this area have found that higher quality LMs (those better able to predict test data) make better predictors of processing difficulty (Frank, 2009; Fossum & Levy, 2012; Goodkind & Bicknell, 2018; Wilcox et al., 2020).^7^ Additionally, recent work comparing architectures has found that surprisal estimates from Transformer-based LMs (Vaswani et al., 2017) tend to be the best predictors of psychometric measures (Hao et al., 2020; Merkx & Frank, 2021; Laverghetta et al., 2022).^8^ Only one recent published study—(Wilcox et al., 2020)—has fit nonlinear GAMs of the linking function using surprisals from a modern Transformer-based LM (GPT-2 Radford et al., 2019).^9^ While they found evidence broadly in favor of a ‘(near-)linear’ linking function, they did not control for by-subject differences. Also, the surprisals they use are from versions of GPT-2 trained on much smaller datasets than the standard pretrained versions, and they do not provide the model with access to context outside of the current sentence. We will compare their results with ours in Discussion.

### Theoretical Arguments for Linearity

A number of lines of work have given theoretical arguments in favor of a linear linking function between processing time and surprisal. Hale (2001) gave the original suggestion that processing effort was proportional to the log ratio of prefix probabilities,^10^ which is equal to surprisal:

$$\begin{array}{l} \text{Time}\left(w_{n}\right)\propto \log\frac{p\left(w_{1:n-1}\right)}{p\left(w_{1:n}\right)}\\ \;=\log\frac{1}{p\left(w_{n}|w_{1:n-1}\right)}=I\left(w_{n}\right) \end{array}$$ (9)

Levy (2005, §2.2.1), showed that the surprisal of a word is equal to the relative entropy between distributions over structures (such as parses, or meanings) before and after observing the word,

$$I\left(w_{n}\right)=D_{\text{KL}}\left(p\left(\cdot |w_{1:n}\right)\left\|p\left(\cdot |w_{1:n-1}\right)\right.\right)$$ (10)

assuming (crucially) that the structures consist at least in part of the words themselves. This provides an additional justification for surprisal theory, linking the processing difficulty of a word to a quantification of the amount by which the comprehender’s beliefs must be updated to account for the observation. The relative entropy between such distributions appears in a number of theoretical analyses of algorithm runtime in Bayesian statistics, notably in the analysis of rejection sampling (Freer et al., 2010) and importance sampling (Agapiou et al., 2017; Chatterjee & Diaconis, 2018; Sanz-Alonso, 2018). However, in both cases the relationship between relative entropy and algorithm cost (number of samples needed) is exponential rather than linear. We are not aware of the analysis of any algorithm that leads to a linear relationship.

Other arguments for the linear linking function come from work which models the comprehender as a rational agent managing the cost of perceptually discriminating between possible alternatives, or preparing resources (Bicknell & Levy, 2010, 2012; Smith & Levy, 2008a, 2008b, 2013). We will not review these arguments here; see Levy (2013) for more detail. In the context of our discussion, the important thing about all such arguments is that they are *computational-level* (in the sense of Marr, 1982). That is, they show that—subject to certain constraints—an optimal information processor would have cost that is linear in surprisal. However, none of these arguments provides a concrete algorithm for achieving this optimal behavior in practice.

### Superlinearity in Surprisal Theory

A number of earlier theoretical proposals have assumed a superlinear linking function between surprisal and processing time. For instance, Narayanan and Jurafsky (2004) conjectured that reading time is inversely proportional to incremental probability—that is, exponential in surprisal.

$$\text{Time}\left(w_{n}\right)\propto \frac{1}{p\left(w_{n}|w_{1:n-1}\right)}=e^{I\left(w_{n}\right)}$$ (11)

Their justification for this linking function is based on a similar intuition to that of Hale (2001), but without assuming the logarithmic relationship. We note this relationship is also the one implicitly assumed by studies using linear models of log-transformed reading times (as in Aurnhammer & Frank, 2019; Boston et al., 2008; Merkx & Frank, 2021; J. Mitchell et al., 2010; Oh et al., 2022; Oh & Schuler, 2023a, 2023b; Roark et al., 2009).

Although much subsequent work has assumed a linear linking function, some of the earliest work in surprisal theory (Levy, 2005, §2.8.8) provided an argument for a *nonlinear* function, motivated by the uniform information density hypothesis (UID; see Jaeger, 2006; Levy & Jaeger, 2006). While the argument itself does not suggest an algorithm, and thus is not relevant to the present discussion, Meister et al. (2021) followed up on the suggestion, experimenting with a linking function of the form

$$\text{Time}\left(w_{n}\right)\propto {\left(I\left(w_{n}\right)\right)}^{k}$$ (12)

where the parameter *k* was fit empirically. They report that their results are consistent with a somewhat superlinear linking function (*k* slightly larger than 1), when using surprisal estimates from high-quality pre-trained Transformer-based LMs.^11^

Models of sentence processing within the ACT-R framework (adaptive control of thought–rational; Anderson & Lebiere, 1998) also make claims about the relationship between the statistical properties of words and incremental processing times. In this framework, an item (such as a word) is recalled in an amount of time that is a function of its *activation*, *A*, as *Fe*^−*fA*^, where *F* > 0, *f* ≥ 1 are parameters. The activation, in turn, is assumed to model the log-odds of the item being needed (Anderson, 1991, simplifying slightly). In accounts of sentence processing within this framework (such as Dotlačil, 2021; Engelmann, 2016; Engelmann et al., 2019; Jäger et al., 2015; Lewis & Vasishth, 2005; Nicenboim & Vasishth, 2018; Vasishth & Engelmann, 2021; Vasishth et al., 2019), the latency formula is taken as an assumption of the model, rather than being explicitly motivated by the intrinsic properties of an algorithm. It is worth noting, however, that the original work proposing this formula did in fact provide a way the formula could be related to the runtime of a serial search algorithm, which we discuss below in Deterministic Search Algorithms. Transforming the ACT-R latency formula from its usual form given above into a statement about surprisal rather than log odds^12^ gives the following superlinear function of surprisal.

$$\text{Time}\left(w_{n}\right)=F{\left(e^{I\left(w_{n}\right)}-1\right)}^{f}$$ (13)

When *f* = 1, as is often assumed, the latency formula then becomes simply the statement that retrieval time increases exponentially in surprisal.

Finally, other recent empirical work which may suggest superlinearity comes from van Schijndel and Linzen (2021) and subsequently Arehalli et al. (2022) who look at reading times in garden-path sentences. They fit linear models of the relationship between surprisal and reading time, and find that these models consistently underpredict the amount to which humans slow down in the critical region. This work is framed as challenging the assumption that reading time can be predicted solely based on incremental surprisal, but an additional interpretation of their results may be that the linking function is superlinear.^13^ Results such as these also highlight the importance of using data with a broad range of surprisal values, since the items with high surprisal will be the most useful in distinguishing whether the shape of the linking function is linear or superlinear.

## EMPIRICAL STUDY

In the preceding sections, we argued that no existing theory of sentence processing provides an algorithmic explanation for processing scaling surprisal, and that a natural class of algorithms that do scale in surprisal are those based on sampling. However, these algorithms predict processing times that are superlinear in surprisal, in contrast to most of the existing literature on surprisal theory, which proposes the relationship is linear and generally assumes constant variance. Additionally, we identified a number of potential problems with earlier empirical analyses which found evidence of a linear linking function. All together, this motivates a re-examination of the empirical relationship, which we present in this section.

We use generalized additive models to predict reading times on the Natural Stories corpus (Futrell et al., 2021), using surprisal estimates from a variety of pre-trained language models, including modern Transformer-based models. In our modelling we control for nonlinear by-subject differences, and allow the effect of surprisal on variance in reading time to be fit empirically. We give a quantitative assessment of the superlinearity of the effect surprisal has on reading time and on variance in reading time.

### Language Models

To get estimates of incremental surprisal values, we use causal^14^ language models (LMs)—statistical models of the probability of words given previous context. An LM *M* gives an estimate of surprisal as I_*M*_ := −log *p*_*M*_(*w*_*n*_|*w*_1:*n*−1_). We obtain surprisal estimates from a collection of LMs, listed in Table 1. These include the following pre-trained Transformer-based LMs: Transformer-XL (TXL; Dai et al., 2019), GPT-2 (Radford et al., 2019), GPT-Neo (Black et al., 2021), GPT-J (Wang & Komatsuzaki, 2021), and GPT-3 (Brown et al., 2020). We also include two older, non-Transformer-based LMs: an LSTM-based model (Gulordava et al., 2018) and a Kneser-Essen-Ney smoothed 5-gram model (both from Boyce & Levy, 2020).

**Table 1.** Language Models used in this study, along with their max context size, number of trainable parameters, amount of pretraining data, and log perplexity score on Natural Stories corpus. For OpenAI GPT-3 models, estimates (marked *) are deduced from evaluations (Gao, 2021).

**model**	**context (tokens)**	**parameters**	**pretraining**	**log PPL**
5-gram	5		90Mtok	6.4
LSTM	NA		90Mtok	4.9
Transformer-XL	NA	88M	100Mtok	4.2
GPT-2	1024	124M	40GB	3.4
GPT-2 large	1024	774M	40GB	3.0
GPT-2 XL	1024	1.5G	40GB	2.9
GPT-Neo	2048	2.7G	800GB	2.8
GPT-J	2048	6G	800GB	2.6
GPT-3 Ada	2048	*350M	300Gtok	3.0
GPT-3 Curie	2048	*6.7G	300Gtok	2.6
GPT-3 Davinci	2048	*175G	300Gtok	2.3

#### Context amount.

One of the main benefits of modern LMs is their ability to incorporate information from large amounts of previous context when making predictions. Different models allow differing amounts of preceding context (Table 1, second column), and for the most accurate estimates of next-token probability, we provide each LM as many previous tokens as it can use. Since all ten stories in the corpus are between 1024 and 2048 GPT tokens in length, this means GPT-Neo, GPT-J and GPT-3 models will always have access to all preceding context in the story when making their predictions. For comparison, we also compute surprisals for each Transformer-based LM when provided only the tokens within the same sentence as the current token. In discussing results below, when we need to distinguish between the surprisals estimated from the same LM with differing amounts of context, we will refer to “within sentence” versus “maximum”-context surprisals. Restricting the amount of context can have a noticeable deleterious effect on language modelling accuracy.^15^

#### Model quality.

To quantify language model accuracy we use *perplexity*—the standard measure of how well an LM predicts a test corpus. The logarithm of perplexity is the mean surprisal, the average uncertainty per word.

$$\begin{array}{l} {\text{PPL}}_{M}\left(w_{1:N}\right)={\left[\prod_{n=1}^{N}\frac{1}{p_{M}\left(w_{n}|w_{1:n-1}\right)}\right]}^{\frac{1}{N}}\\ \log\;{\text{PPL}}_{M}\left(w_{1:N}\right)=\frac{1}{N}\sum_{n=1}^{N}I_{M}\left(w_{n}\right) \end{array}$$

A lower perplexity language is one which can more accurately predict tokens given previous context. Note, the perplexity of two models is not directly comparable unless they have the same vocabulary. All eight GPT-type models we use are directly comparable.^16^ The remaining three models (the LSTM, *n*-gram, and Transformer-XL) are not. For this reason, while we will use perplexity values for all models in discussion and figures to follow, we will only make direct comparisons of the GPT models.

### Corpus

For our empirical analysis we use the Natural Stories corpus (Futrell et al., 2021), an English-language corpus which was released with self-paced reading time (RT) psychometric data. The corpus consists of 10 stories, of about 1000 words each. Each story is a modified version of publicly available text, edited to contain “many rare or marked syntactic constructions, … while maintaining a high degree of overall fluency and comprehensibility.” The relatively high concentration of rare constructions makes this corpus particularly appropriate for our study, since the difference between a linear and a superlinear linking function may only be appreciable in the high end of the surprisal range. Reading times released with this corpus were gathered from 181 native speakers, with each word in the corpus read by a median of 84 reading participants.

To allow inspection of the full text of the corpus, annotated with LM surprisals and reading times, we provide an interactive utility, linked in Appendix E.

### Generalized Additive Models

We fit GAMs to model the effect of surprisal on reading time. In particular, we use Gaussian location-scale mixed models (Rigby & Stasinopoulos, 2005; Wood et al., 2016) which allow us to model surprisal’s nonlinear effect on mean RT, while also modelling its nonlinear effect on variance in RT, rather than assuming variance is constant or has a particular parametric relationship to the mean.

For each LM’s set of surprisals, we fit a model we will call the **nonlinear GAM**, which predicts reading time, and variance in reading time (in the form of log standard deviation), each as an overall nonlinear function of surprisal, controlling for nonlinear by-subject variation and control predictors. It is these nonlinear GAM fits which we will use to interpret the relationship between surprisal and reading time. We also fit a minimally-different control model for each LM’s surprisals, which we will call the **linear control GAM**, in which overall and by-subject effects of surprisal (for predicting both reading time and variance in reading time) are forced to be linear.

#### Model specification.

In specifying the nonlinear GAMs, we include the following terms for the effect of surprisal and control predictors. To model the linking function we are interested in, we include a smooth term for the overall nonlinear effect of surprisal. To control for possibly nonlinear individual deviations from the overall curve, we include a by-subject factor-smooth interaction term. We also include a tensor product term for the nonlinear interaction between log-frequency and word length (following Goodkind & Bicknell, 2018; Smith & Levy, 2013; Wilcox et al., 2020). Finally we include versions of all three above terms but for the previous word, to control for spillover effects (following Goodkind & Bicknell, 2018, 2021; Meister et al., 2021).

To predict variance (precisely, log standard deviation) in reading time, we include the same terms as above, though only for the current word, since there is no a priori reason to expect spillover in variance. So that the resulting overall curve fit by the model can be interpreted simply, we choose a relatively low number (*k* = 6) for the basis dimension, effectively limiting the maximum wiggliness of the fitted curve.

For the linear control GAMs, we use the same model specifications as for the nonlinear GAMs above, but with the main surprisal smooth and factor-smooth interaction terms replaced with a linear parametric term and linear by-subject random effects (likewise for the previous word, and for the effect on variance). To differ only minimally from the nonlinear GAMs, we allow the terms for the interactions between length and frequency to remain nonlinear similar to the approach taken in Goodkind and Bicknell (2018).

We give further details and discussion of the specification of GAMs in Appendix C.^17^

## RESULTS

**Figure 2.**
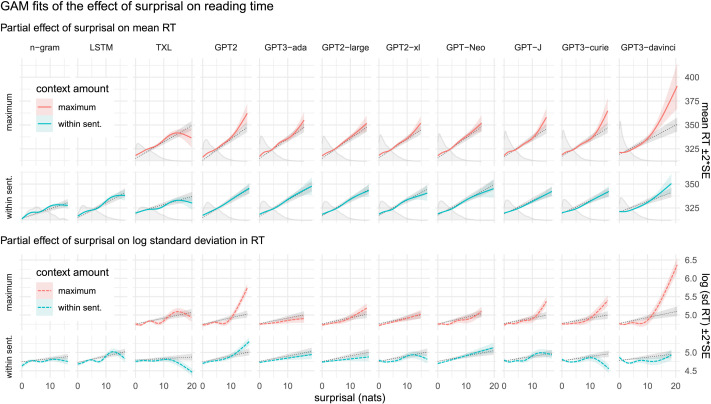
**The effect of surprisal on self-paced reading time.** Colored lines are the fitted effects from the nonlinear GAMs, dotted black lines beneath are from the corresponding linear control GAMs. **Top two rows**: effect of surprisal on mean RT, with density plots of surprisal underlaid at the bottom. The top row (red) uses surprisals from LMs with full access previous context, the second row (blue) uses LMs with access only to within-sentence context. **Bottom two rows**: as the first two, but for the effect of surprisal on variance in RT (as log standard deviation). Shaded regions represent 95% CIs.

Figure 2 displays our main results, showing the relationship between surprisal and human reading time for each LM and context amount. Each curve represents the nonlinear GAM’s fitted effect of surprisal on mean RT (top two rows, solid colored lines), or on log standard deviation in RT (bottom two rows, dashed colored lines). In each small plot, the linear linking function predicted by the corresponding linear control GAM is underlaid as a black dotted line. Density plots at the bottom of each plot for the mean effect show the distribution of that LM’s estimated surprisal values. The curves for LMs with maximum context are plotted in blue (first and third rows); within-sentence context in red (second and fourth rows). LMs are ordered left-to-right by decreasing perplexity, given maximum context.

We first examine the effect of surprisal on RT (top set of plots). For all language models, reading time generally increases with surprisal. Impressionistically, better LMs (as measured by perplexity) appear to exhibit a superlinear relationship between surprisal and reading time, with higher quality LMs exhibiting more strongly superlinear curves (see below for quantification of this claim). By contrast, lower quality LMs (including the *n*-gram, LSTM, Transformer-XL), and models with only within-sentence context, tend to exhibit closer to linear relationships—or even *sub*linear relationships at high surprisal values (see Discussion). The slopes fit by the linear control GAMs are positive for all models.

Examining the relationship between surprisal and variance (as log standard deviation; bottom set of plots), we see a similar pattern. Variance in RT appears to generally increase with surprisal, with a few exceptions among the models with only access to within-sentence context. And for the linear controls, we generally see a positive slope for all fitted lines, similarly to the slopes fit by these control models for the effect on RT.

### Quantifying the Direction of the Effect

**Figure 3.**
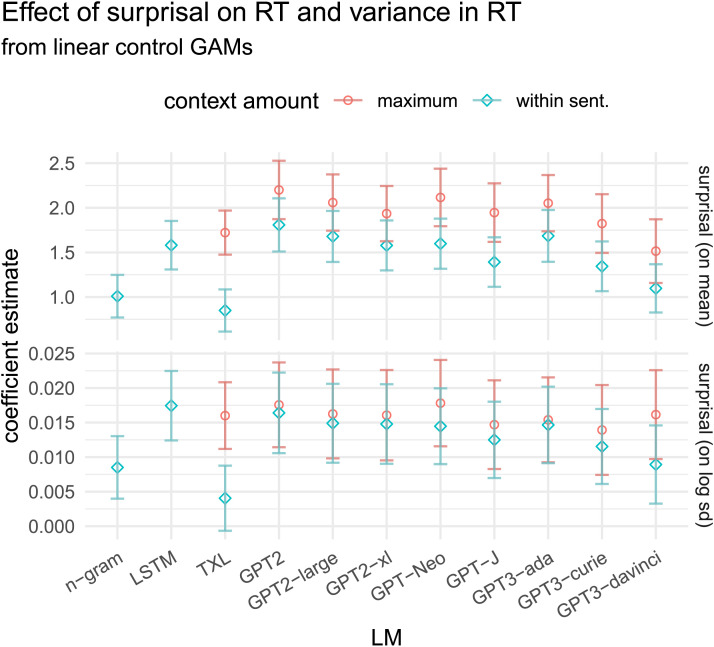
**Coefficient estimates (with 95% CI) for the main effect of surprisal on RT and log standard deviation in RT, as fit by the linear control GAMs.** For all LMs, both coefficients are positive, and significant (*p* < 0.05)—with the exception of the variance effect for Transformer-XL constrained to within-sentence context.

To establish the overall direction of the effect, as well as replicate earlier work which used linear models for the effect on RT (though not variance), we will start by examining the slopes fit by our linear control GAMs. We use these models to get a quantitative interpretation of the overall direction of these effects, before introducing our superlinearity measure to examine the shape of the curve in the next subsection. Figure 3 provides the coefficients for the effect of surprisal. Each point describes the slope of the relationship between surprisal and RT (top) or log standard deviation in RT (bottom), with bars indicating 95% confidence intervals.

We observe that surprisal has a positive effect on RT for all LMs, consistent with the findings of the large number of previous studies of this relationship. This is also true for variance in RT: As surprisal increases, variance in reading time also increases, for all LMs and context amounts.^18^ This is noteworthy, given that previous work has nearly universally assumed that variance is constant. Incidentally, we also note a general trend that the effect of surprisal on mean RT is larger when using LMs with access to full previous context compared to restricting to only within-sentence context,^19^ though this is not true for the effect on variance in RT (with the exception of Transformer-XL).

### Quantifying Superlinearity

To quantify the observation that the relationship seems more superlinear for better quality LMs, we define a simple descriptive value which we will call *superlinearity*. This value is computed as follows: (i) split the surprisal range into two equal intervals, (ii) find the slope of the best linear approximation to the curve in each interval, and (iii) take the difference between these two slopes. A curve which bends upward will have positive superlinearity; one which bends downward will have negative superlinearity. For a relationship which is overall increasing^20^ positive superlinearity indicates that the curve is increasing superlinearly in a global sense, though it may not be locally monotonic.

**Figure 4.**
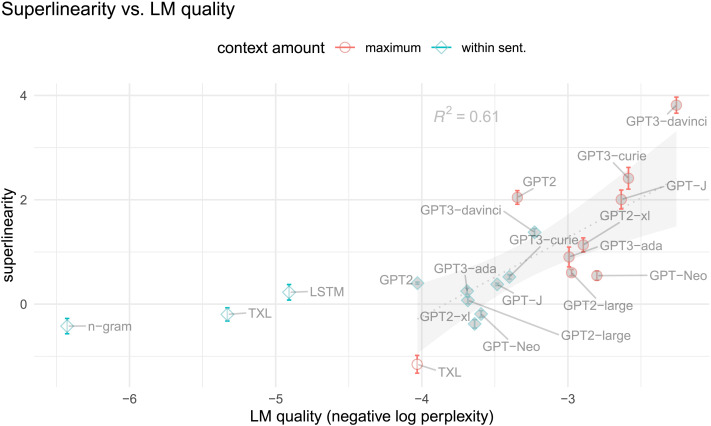
**Superlinearity, measured as the amount by which the slope of the nonlinear GAMs’ predictions at high surprisal exceeds that at lower surprisal, versus model quality (as negative log perplexity).** The effect of surprisal on reading time is more superlinear for better LMs, as demonstrated by a best-fit regression line (dashed line with 95% CI shaded and correlation coefficient *R*^2^ printed above). Note only GPT-based models (filled grey) are directly comparable by perplexity, hence the line describing this trend is fit on only those points.

Figure 4 presents superlinearity plotted against LM quality (as negative log perplexity, so that higher values represent better LMs). Points for GPT-based models—which share a common tokenization scheme and vocabulary and are thus directly comparable by perplexity—are filled in grey, and a weighted linear regression fit on these points is displayed as a dashed line, with correlation coefficient printed above, and 95% CI shaded.

We see a clear correlation between an LM’s quality and the superlinearity of the effect on RT. This correlation is evident visually, and is attested by the correlation coefficient *R*^2^ = 0.61. This provides a quantitative confirmation of our claim that the better the LM, the more superlinear the effect of surprisal on reading time.

### Controls

In our modelling we chose to fit the effect of surprisal on variance, unlike previous work, which has often assumed constant variance. To check whether the superlinearity we see in the relationship with mean RT is dependent on this modelling choice, we fit models which assume constant variance. For this control, we assume a normally-distributed dependent variable and identity link (as is standard, following Goodkind & Bicknell, 2018; Smith & Levy, 2013; Wilcox et al., 2020).^21^ We found the relationships between surprisal and RT predicted by these models were similar to the results reported above. They exhibited increasing nonlinearity with increasing LM quality (plots from these models, and further details, are in Appendix G).

In our models, we controlled for spillover effects by including predictors for one previous word (following e.g., Goodkind & Bicknell, 2018, 2021; Meister et al., 2021). However, other studies (including Smith & Levy, 2013) have argued for using up to 3 previous words. To understand whether this choice is likely to have influenced our general results, we include additional analyses in Section 12, examining autocorrelation in residuals and fitting models with predictors for three previous words, rather than one. We find there is little evidence to suggest that additional spillover predictors would have a large effect on our main qualitative results.

In order to understand the degree to which our results are dependent on nonlinear by-subject effects we include, we experimented with fitting models as above, but in which we removed the terms controlling for by-subject effects. We found that this modification resulted in predicted relationships that were similar in shape, but with much wider confidence intervals. This suggests that controlling for by-subject variation in this data gives us higher power to detect population-level nonlinear effects. This control is also useful for comparing our results with previous literature which did not include by-subject random effects (e.g., Fernandez Monsalve et al., 2012; Hofmann et al., 2022; Smith & Levy, 2013; Wilcox et al., 2020). Not controlling for by-subject variation may be one reason why such studies did not find evidence of a nonlinear effect.

As is readily evident in the density plots of surprisal values (plotted in Figure 2, top two rows), the overwhelming majority of words have relatively low surprisal. This is especially true for the lowest-perplexity LMs. To check that the shape of the curves we see are not being determined by a few high-surprisal outliers, we carried out two controls. First, we carried out a cross-validation, refitting GAMs for each of the LMs on 6 folds of the data.^22^ We found that the degree of superlinearity in the results was consistent across folds, confirming that the results are not driven by a small number of outliers (see Appendix G). Second, focusing on the most superlinear GAM, which also has the most drastically skewed distribution of surprisals (GPT-3 Davinci), we performed a hand-inspection of the highest-surprisal words, and found that most occur within the kinds of rare syntactic examples that Natural Stories was designed to contain, but otherwise seem plausible in context, and therefore do not seem to be outliers in any way which should have required their removal from our data (see Appendix F for a complete list of these words in context and further discussion). We then re-fit GAMs with the highest surprisal items removed. We found that superlinearity was somewhat reduced (due to truncating the range of surprisals), but curve remained superlinear.

## DISCUSSION

In the first part of this paper, we investigated the runtime characteristics of inference algorithms that iteratively sample from the prior—a natural example of a broad class of algorithms whose runtime scales with surprisal. As we showed, simple examples of such algorithms predict that both runtime and variance in runtime increase with surprisal, the former superlinearly. In the second part, we carried out an empirical study to test these predictions, finding that for one widely-studied dataset the empirical relationship between surprisal and processing time is broadly consistent with these predictions when using the best-available LMs to estimate surprisal. In this section we discuss the implications of these results.

The correlation we observe between LM quality and superlinearity suggests that one reason why a superlinear relationship has not been detected in earlier work may simply be due to the use of surprisal estimates from earlier language models, which were less accurate. For example, as discussed in Empirical Studies in Surprisal Theory, Smith and Levy (2008a, 2013) found empirical support for the linear linking function, using a trigram model to estimate surprisal. Our results confirm their finding for this type of LM, showing no evidence of superlinearity for the *n*-gram model. Wilcox et al. (2020) also presented evidence of a linear linking function, using some higher quality LMs and multiple datasets, including the Natural Stories corpus. However, their highest-quality LM was a GPT-2 model trained on much smaller datasets than the pretrained GPT-2 model we use,^23^ and they estimate surprisals using only within-sentence context. Both choices likely mean less accurate predictions in general (higher perplexity), although they do not report perplexity values. As our results demonstrate, using LMs restricting to only within-sentence context, and using higher-perplexity LMs in general, tends to reduce the superlinearity of the relationship.

**Figure 5.**
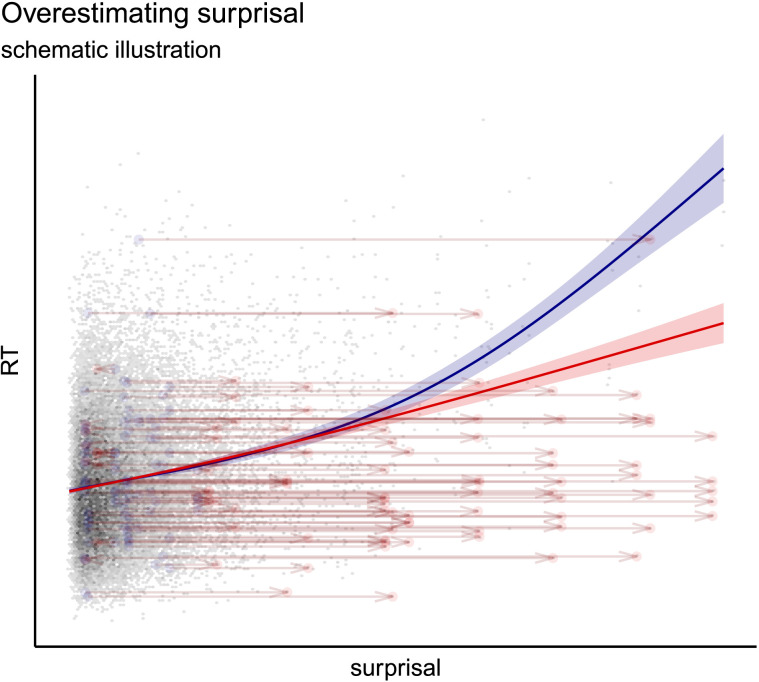
Diagram illustrating schematically how a superlinear relationship may look linear if some surprisal values are systematically overestimated: When a subset of points are moved higher in the surprisal range (indicated by arrows), the best-fit curve becomes less superlinear (blue to red).

This tendency is consistent with the following interpretation, illustrated schematically in Figure 5. The blue curve represents the best-fit curve for reading time as a function of hypothetical ‘true’ surprisal, and the red curve represents the best-fit curve after raising the surprisal values assigned to a subset of observations (while keeping their reading times the same). A lower quality (higher perplexity) language model will tend to overestimate surprisal in general (since log perplexity is simply average surprisal). If an LM consistently overestimates surprisals compared to humans in such a way, we would expect the resulting best-fit linking function to be lower than it should be at the higher end of surprisal range, due to these items with low reading time being (wrongly) assigned high surprisal.^24^ As illustrated in the diagram, such underestimation (moving these points rightward) results in changing the best-fit curve from superlinear (blue), to linear (red). This is what we see in our results; the lower quality LMs display less superlinear relationships (or even sublinear ones in some cases, especially those restricted to only within-sentence context). Under this interpretation, the superlinearity we observe in our results stems from our using more accurate surprisal estimators and, in particular, models which can make best use of large amounts of previous context to accurately predict words.

An additional factor that may explain why superlinearity has not been observed in previous studies that fit GAMs to describe this relationship is that most did not control for by-subject variation (Hofmann et al., 2022; Smith & Levy, 2013; Wilcox et al., 2020), or assumed that such variation could be modeled by a constant offset (Goodkind & Bicknell, 2018). As described in the previous section, our experiments lesioning the by-subject random effects from our GAMs resulted in models which were much less confident about the shape of the curve, even for the more accurate LMs.

As mentioned in Superlinearity in Surprisal Theory, a recent line of work introduced in van Schijndel and Linzen (2021) has examined garden path effects, where humans show increased processing difficulty at the point in a sentence where temporary structural ambiguities are resolved in favor of the less expected alternative. Van Schijndel and Linzen (2021) and Arehalli et al. (2022) argue that the degree of slowdown that occurs in humans exceeds that which can be predicted by linear linking function. We propose that intuitively, a superlinear linking function (such as those we see in our results) should be able to predict a larger slowdown the than a linear one, and thereby at least partially explain the human slowdown observed in their study. However, in the current study, our focus is on determining the best-fit form of the linking function broadly. We don’t necessarily predict that the general superlinear trend we see in our results (for GPT-3 Davinci, for instance) should be sufficient to entirely explain the human reading times on particular sentences, where many other factors specific to that particular sentence may influence human reading times. However, with proper controls, examining the degree to which a superlinear linking function can explain human processing on particular grammatical constructions (including garden path sentences) is a promising direction for future work.

### Language Model Perplexity and Quality as Psychometric Models

In this work, we use pre-trained LMs as the best-available approximators of the true predictability of individual words—the quantity which should describe the behavior of an optimally rational comprehender. The interpretation of our results relies on the assumption that more accurate LMs provide better estimators of human surprisal, at least for those words which drive the superlinear fit of our GAMs. As discussed above, this assumption is supported by recent literature (Goodkind & Bicknell, 2018; Hao et al., 2020; Laverghetta et al., 2022; Merkx & Frank, 2021; Wilcox et al., 2020). Very recently, however, another line of work has emerged arguing that, to the contrary, lower perplexity LMs sometimes provide *poorer* fits to psychometric data. Building on a preliminary observation in Oh et al. (2022), Oh and Schuler (2023b) present a study of three different families of Transformer-based LMs (GPT-2, GPT-Neo, and OPT; S. Zhang et al., 2022), finding that the lower-perplexity LMs in each family tend to have poorer psychometric predictive power. In related work, Kuribayashi et al. (2022) report that for GPT-2 and LSTM models, psychometric predictive power increases as access to context is restricted, in English and Japanese. This improvement in psychometric predictive power continues even for extremely severe restrictions such as limiting context to just one previous word.

These studies raise two important problems to be explored in future work. First, it is important to understand which subsets of words drive the two effects (psychometric power and superlinearity) and how much they overlap. If the words driving the decrease in psychometric power are not the same as those driving the superlinearity effect, then these studies and our own may be complementary. For example, Oh and Schuler (2023b) show that named entities and predicative adjectives are among the classes of words most responsible for the decreasing psychometric predictive power. Intuitively, better LMs may underestimate how surprising these items are to people because the LMs are trained on superhuman quantities of data. It is possible for a model to find such words much less surprising than humans, while improving the psychometric fit of other classes of words, such as function words. If the latter classes of words are those most critical for superlinearity, then both effects could very well hold. Determining whether this is or is not the case requires a detailed sensitivity analysis that carefully matches datasets, LMs, and analytical models. We leave this to future work.

A second, and more important, question is whether these recent results are an artefact of using linear models to study the relationship between surprisal and processing time. Our analyses above show that the lower-perplexity a model is, the greater the advantage of a superlinear linking function over a linear one. Studies such as Kuribayashi et al. (2022) and Oh and Schuler (2023b) make use of linear linking functions,^25^ showing that lower perplexity LMs predict psychometric results more poorly. However, if the true relationship between surprisal and processing time is nonlinear, then the seeming decrease in psychometric predictive power that they report might even be related to the increasing superlinearity that we observe. A large-scale examination of the relationship between LM perplexity and psychometric predictive power using nonlinear regression models such as GAMs would provide a useful contribution to more fully understand the potential three-way relationship between LM accuracy, psychometric predictive power, and superlinearity.

### A Particle Filter Model

To our knowledge, the only explicit sampling-based model of incremental sentence processing to date is the approach presented in Levy et al. (2008). Their model uses particle filtering, a standard sequential Monte Carlo (SMC) technique based on importance sampling (Doucet et al., 2001; Doucet & Johansen, 2011). The parsing algorithm estimates the posterior distribution *p*(*z*_*n*_|*w*_1:*n*_) with a collection of *K* weighted particles (partial parses). Each of these particles is first obtained by sampling from the prior *p*(*z*_*n*−1_|*w*_1:*n*−1_). Then each particle is updated according to an incremental transition distribution *p*(*z*_*n*_|*z*_*n*−1_), and weighted proportional to how likely it is to explain the next observation (word): *p*(*w*_*n*_|*z*_*n*_).^26^ Because their algorithm uses a fixed number of particles (the beam width, *K*), the number of samples drawn is identical at every word. Thus, this algorithm’s runtime does not directly depend on surprisal in the way that the algorithms that we examined above do.

However, Levy et al. offer an analysis of processing difficulty which can be related indirectly to the present work. Rather than relating difficulty to runtime via expected number of samples, they relate processing difficulty at a particular word to the probability of failure at that word—that is, the probability that none of the particles in the beam can be extended to explain that word. They estimate this quantity by running the particle filter multiple times and counting the proportion of runs where the set of particles contains no successful parses.

This probability of failure is directly related to our analysis in Simple Guessing Algorithm, where runtime is inversely proportional to the probability of success (one minus the probability of failure). In the particle filter, the probability of success at step *n* is the probability that at least one particle contains a successful parse for *w*_*n*_. If the particles are sampled from the *exact* posterior Pr(·|*w*_1:*n*−1_), the number of such samples required for an accurate approximation of the posterior Pr(·|*w*_1:*n*_) scales as *e*^I(*w*_*n*_)^ = 1/Pr(*w*_*n*_|*w*_1:*n*−1_).^27^ In the particle filtering setup, which estimates the posterior distribution using importance sampling from an *approximate* prior, the expected number of samples required to integrate *w*_*n*_ is at least *e*^I(*w*_*n*_)^.^28^ This suggests that a modified version of the particle filtering model, where variable numbers of samples were drawn until some desired number of successful parses were obtained, would have runtime that scaled naturally in surprisal. Examples of this type of modified approach to particle filtering include adaptive beam width algorithms (such as Buys, 2018; Elvira et al., 2017; Fox, 2003), which allow the number of particles (*K*) to vary at each step in order to maintain a criterion such as a bound on probability of error, or uncertainty of the model. Such algorithms could potentially be natural for use in models of sentence processing, and would have the property that higher surprisal words would require (exponentially) more samples.

### Deterministic Search Algorithms

Besides nondeterministic sampling algorithms, we identified a related class of deterministic algorithms whose runtime scales in surprisal: those involving probability-ordered search.^29^ In particular, probabilistic pruning (where only the high prior-probability parses are kept) has the potential to predict a monotonic increasing relationship with surprisal. Such methods (like beam search; Y. Zhang & Clark, 2008), have seen extensive use in parsing literature (see e.g., Bouchard-Côté et al., 2009; Jurafsky, 1996; Meister, Cotterell, & Vieira, 2020; Meister, Vieira, & Cotterell, 2020; Roark et al., 2009; Vieira & Eisner, 2017), yet as far as we are aware, there are no results relating these specific algorithms’ time complexity to surprisal or incremental probability.

As noted above in Superlinearity in Surprisal Theory, one simple and specific deterministic algorithm which can predict runtime increasing as a function of surprisal is the serial search mechanism assumed in the rational analysis of memory and ACT-R literature (Anderson, 1990; Anderson & Lebiere, 1998). The formula for reaction time in this framework was originally derived under the assumption that items in memory are considered in order of decreasing need probability. If each item requires a fixed amount of time, the runtime is simply the ordinal position of the item in a probability-ordered list.^30^ Using this argument, along with the assumption that item need-odds are power-law distributed,^31^ Anderson and Lebiere (1998) derived the latency formula linking (log) odds to run time exponentially as *Fe*^−*fA*^. We noted above this can be restated as *F*(*e*^I(*w*_*n*_)^ − 1)^*f*^—a superlinear function of surprisal (eq. 13).

The upshot of this analysis (independent of the specifics of the ACT-R framework) is that the runtime of simple probability-ordered search makes a concrete prediction about the linking function with surprisal. And, this prediction is similar to the predictions of sampling algorithms we have discussed. However, unlike the sampling-based mechanisms we explored, a deterministic ranked-search mechanism such as this cannot predict nonzero variance in any intrinsic way.^32^

### Conclusion

In this work, we have considered inference algorithms that involve iteratively sampling from a prior, and proposed that such mechanisms provide a plausible framework for formalizing theories of incremental processing, since their complexity naturally depends on the predictability of their input. Analyzing simple representative examples of this class of algorithms, we found that the number of samples required scales superlinearly as a function of surprisal, with variance also increasing. In our empirical study of human reading times we found evidence of a linking function consistent with these predictions, when using surprisal estimates of the most accurate modern LMs.

## ACKNOWLEDGMENTS

We thank our anonymous reviewers for their detailed comments and suggestions. We also thank Jakub Dotlačil, Michaela Socolof, Benjamin LeBrun, and the members of Montréal Computational and Quantitative Linguistics Lab (MCQLL), and MIT Probabilistic Computing Project (ProbComp) for valuable feedback.

This research was enabled in part by resources provided by Mila (mila.quebec), Calcul Québec (calculquebec.ca) and the Digital Research Alliance of Canada (alliancecan.ca). We also gratefully acknowledge the support of the Canada CIFAR AI Chairs Program, the Centre for Research on Brain Language and Music (CRBLM.ca), the Natural Sciences and Engineering Research Council of Canada, and the Fonds de recherche du Québec - Société et culture (FRQSC).

## Notes

^1^ The particle filter model proposed in Levy et al. (2008) is a specific example of such an algorithm applied to parsing, but due to modelling choices, its runtime doesn’t scale in surprisal. We will discuss this model in A Particle Filter Model.^2^ This simple sequential sampling algorithm, also mentioned in Freer et al. (2010), is sometimes informally referred to as ‘rejection sampling.’ We use the term ‘guessing’ to avoid confusion with the more general rejection sampling algorithm (as defined in, e.g., Chopin & Papaspiliopoulos, 2020, alg. 8.1), of which it is a special case.^3^ This is intentionally the simplest possible version of such an algorithm. Among the many possible refinements (which might be sensible in practice) would be to continue guessing until some reasonable number of successes, rather than stopping at the first success. Note that such a modification does not change the asymptotic complexity, simply adding a constant multiplier. As noted above, we do not analyze such particular modifications since we are not proposing a specific algorithm. Our goal with these analyses is to understand the general asymptotic complexity characteristics of the class of algorithms which involve iterative guessing from the prior.^4^ Note the probability Pr(*i* ≺ *j*) depends on the weights of items *i* and *j*, and no others. This means it is independent of the order the other items are drawn in, what their probabilities are, and even whether drawing is done with or without replacement.^5^ Others have used linear models with a log-link, or log-transformed dependent variable (e.g., Aurnhammer & Frank, 2019; Boston et al., 2008; Merkx & Frank, 2021; J. Mitchell et al., 2010; Oh et al., 2022; Oh & Schuler, 2023a; 2023b; Roark et al., 2009), implying an exponential relationship between surprisal and reading time (see Superlinearity in Surprisal Theory).^6^ Smith and Levy (2013) did examine the nonlinear effect of surprisal on fixation time for eye-tracking data, fitting nonlinear GAMs for each subject separately, but not as random effects in a common model, and not for self-paced reading data, due to lack of a sufficient data to fit such models.^7^ However, some very recent work has begun to argue the opposite—that higher perplexity LMs or those using only limited context may be better psychometric models (e.g., Kuribayashi et al., 2022; Oh & Schuler, 2023a; 2023b). We will return to this topic in Discussion.^8^ Note, these studies mostly implicitly assume a linear relationship, using *χ*^2^ or linear models’ difference in log likelihood to assess psychometric predictive power.^9^ In recent unpublished work, Shain et al. (2022) conduct a new large-scale study of the linking function using multiple LMs, including modern pretrained Transformer-based models, using nonlinear continuous-time deconvolutional regressive neural networks (CDRNNs; Shain & Schuler, 2022), rather than GAMs. We discuss their results and preliminarily compare with ours in Appendix D.^10^ Hale assumed prefix probabilities according to a probabilistic context-free grammar Earley parser, but this is not crucial to the intuition.^11^ Cf. Brothers and Kuperberg (2021) who recently presented evidence for a *sub*linear linking function, using cloze-probabilities (Taylor, 1953), not LMs, to estimate surprisal. Note however, cloze probabilities are in practice impossible to estimate for high-surprisal items (see Levy, 2008a; Smith & Levy, 2011), and LM surprisals generally give an empirically better fit to psychometric data (Hofmann et al., 2022).^12^ Via the identity log odds(·) = −log(*e*^−log *p*(·)^ − 1). We believe we are the first to note this way of relating ACT-R’s latency formula with surprisal theory.^13^ Note this interpretation does not necessarily contradict their framing, provided the human slowdowns they observe are larger than even the best-fit superlinear linking function could predict—see Discussion.^14^ We only consider unidirectional or causal LMs: models which predict words given previous context, without access to future context. Bidirectional or masked LMs are less appropriate for modelling incremental processing.^15^ Note however that some recent work has suggested that restricting context can increase psychometric predictive power: See discussion in Language model perplexity and quality as psychometric models.^16^ All use the byte-level BPE tokenization scheme of GPT-2 (Radford et al., 2019).^17^ Scripts for data preprocessing and reproducing all results and figures are available at github.com/mcqll/plausibility-sampling-processing.^18^ These coefficients are all significantly different from zero (at the 0.05 level), with the sole exception being Transformer-XL when only given within-sentence context, for which the coefficient is positive but not significant.^19^ However, this difference is only significant for TXL, GPT-Neo and GPT-J (at the 0.05 level)—for all the other models the difference is just barely beneath this threshold for significance.^20^ Note that this definition of superlinear doesn’t imply increasing—a U-shaped curve would be superlinear. This is a reason for the previous analysis showing all effects were increasing.^21^ The assumption of constant variance could also be relaxed by only partially, by assuming a specific parametric relationship between mean and variance. See details in Appendix C.^22^ We also note that the evaluation technique used to fit GAMs is designed to control against such sensitivity to outliers (see discussion in Wood, 2011).^23^ They use versions of GPT-2 trained on multiple different datasets, with the best model they use being trained on 42 million tokens, compared to the ∼40 GB (roughly 10 billion tokens) of training data for the GPT-2 model which we use.^24^ One way this may occur for an LM with restricted access to context, for instance, is when it it consistently assigns high surprisal to see some uncommon words in a text where, given the context, they are not surprising to humans, who have a good model of the topic being discussed.^25^ Though this picture is complicated by differing choices on whether to log-transform the reading times before fitting models (as discussed above): we do not transform, nor do Kuribayashi et al., while Oh and Schuler do. Note, Shain et al. (2022) also observe that GPT-2 performs better than GPT-3 and GPT-J overall, though their study is aimed at determining the shape of the linking function, not the relationship between perplexity and psychometric power—see Appendix D for further discussion.^26^ The algorithm is recursive, so the representation of the prior *p*(*z*_*n*−1_|*w*_1:*n*−1_) is itself an estimate of the posterior from the prior step, computed using samples from *p*(*z*_*n*−2_|*w*_1:*n*−2_), etc.^27^ This can be seen by first recalling that surprisal equals the relative entropy between prior and posterior (Levy, 2005)—again, assuming that the full parses consist at least in part of the words themselves. Then, note that in importance sampling, the number of samples required for accurate estimation scales as the exponent of precisely this relative entropy (see Chatterjee & Diaconis, 2018, Thm. 1.2, also discussed in Agapiou et al., 2017; Sanz-Alonso, 2018).^28^ Given the approximate prior makes predictions that are on average no better than the true prior, the expected number of samples will be no smaller than the expected number from the true prior.^29^ This is not necessarily a separate class of algorithms in any discrete sense, but rather may potentially be viewed as a special subset of sampling algorithms, since any deterministic algorithm can be framed as sampling from delta functions.^30^ The original argument (Anderson, 1990, ch. 2) predated ACT-R. A modified version for ACT-R, which is stated in terms of activation rather than need probability is given in Anderson and Lebiere (1998, app. 3B).^31^ This assumption is very similar to our assumption that item weights are Pareto-distributed, in our analysis in Guessing Without Replacement Algorithm.^32^ In the ACT-R framework, in practice, a noise term is added to the basic latency formula, but this is not motivated by the deterministic search algorithm used to derive the basic formula.^33^ After removing words with different tokenizations, 91% of tokens remain for the *n*-gram and LSTM, 80% for Transformer-XL, and 78% for the GPT models.^34^ The factor smooth interaction basis we use fits a nonlinear random effect for each subject (with a TPRS basis and basis dimension *k* = 10, by default). The key point is that using factor smooth rather than random slopes, not which exact factor smooths used, which matters less (as explored in Sóskuthy, 2021).^35^ A smooth term s(x, g, bs='re') for the random effect of variable x with grouping factor g encode a random effect of x for each level of g, but not by-group means. Adding random intercepts in separately, with an additional term s(g, bs='re') will encode an assumption that all slopes and intercepts are independent (see Wood, 2017, §3.5.2).^36^ However, as Wood notes, this technique is generally unnecessary when the smoothing parameter is efficient to fit, as a smooth would be automatically shrunk to linear if the data merit it.^37^ A model with a gamma-distributed response (as used by, e.g., Hofmann et al., 2022) has this property. This is likewise true for inverse Gaussian, or even log-normal models, though the specific assumption is different in each case (see Lo & Andrews, 2015 for a discussion of these choices for modelling reading-time data with generalized linear models). An assumption of a inverse Gaussian or gamma distribution would also potentially be a principled choice for an underlying process involving sampling, given these distributions model waiting time.^38^ With an unconstrained (nonlinear) linking function this is less clear: GPT-J does not underperform GPT-2, but GPT-3 does. However, we note this trend reverses in their results when considering just the self-paced reading datasets in their study. In fact fully nonlinear GPT-3 and GPT-J perform better than GPT-2 for self-paced reading data from both available corpora (Natural Stories and Brown).^39^ Difficulty in ORCs has been explored in a number of previous studies focusing on predictions about where the locus of difficulty is—the subject or the verb, with the former traditionally being the prediction of surprisal-based theories (see e.g., Levy et al., 2013; Staub, 2010; Traxler et al., 2002; Vani et al., 2021). It is perhaps interesting to note that words from *both* critical places in ORCs are represented in the list of highest-surprisal items—not just at the subject, but also at the verb.^40^ We attempted fitting models with more previous words (*M* = 3 and *M* = 4), but found that this resulted in models whose design matrices that were too big for mgcv::gam. Unfortunately the more efficient procedure bam is not currently implemented for location-scale GAMs.^41^ For lag *k*, the autocorrelation function ACF(*k*) gives the correlation between observations *k* words apart; partial autocorrelation PACF(*k*) is the amount of correlation that is not accounted for by lags 1 through *k* − 1.

## Figures and Tables

**Figure B1.**
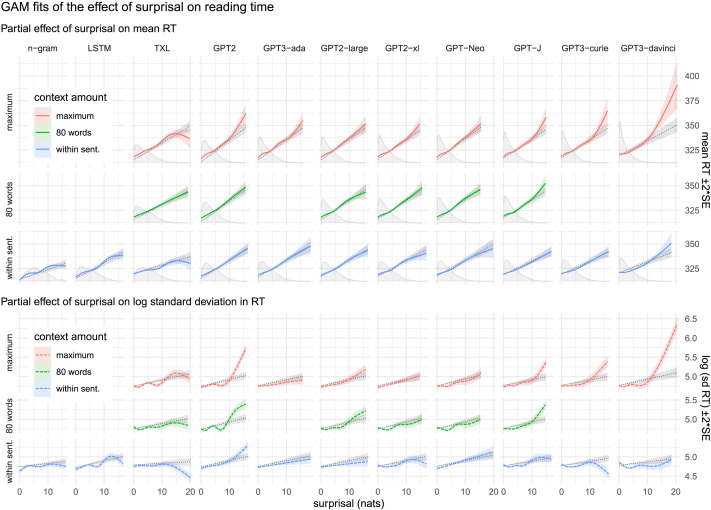
Plots of all GAM models repeated from Figure 2, with the addition of select LM models with 80 previous words of context (middle row, green) as a middle-ground between maximum context and within sentence context.

**Figure B2.**
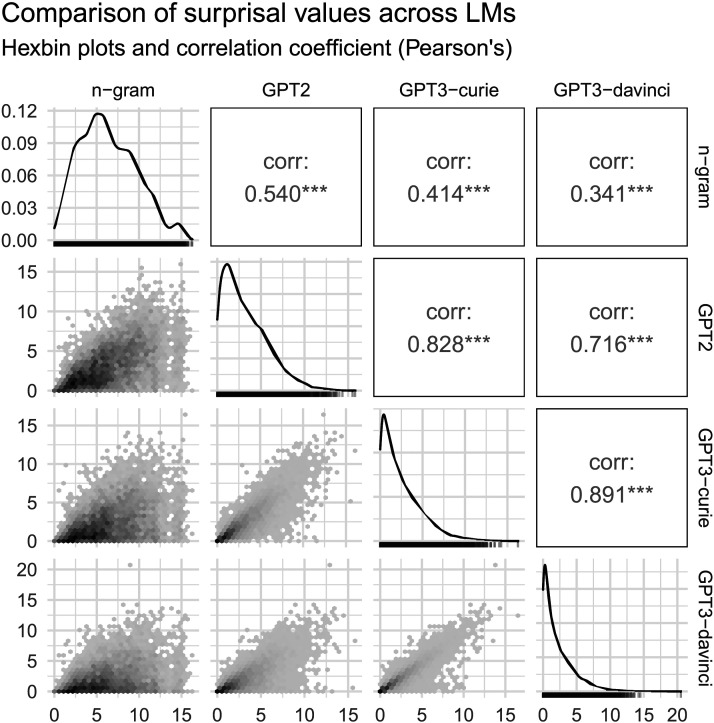
**Comparison of surprisal estimates from a selection of language models, by item in the corpus, with density and rug plots for each LM on the diagonal.** Pearson’s correlation coefficients for each of the pairs are given in the upper right.

**Formula 1.**
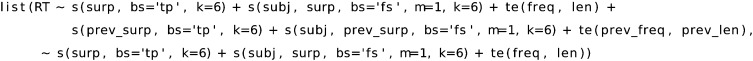
The mgcv formulae used for the **nonlinear GAM** fits. RT is predicted as a nonlinear function of surprisal, controlling for nonlinear by-subject effects, and interactions between frequency and word length. The mean formula also includes similar predictors previous word as well as current, to control for spillover effects.

**Formula 2.**
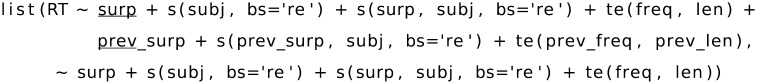
The formulae used for **linear control GAM** fits. The interpretation is effectively the same as that of formula 1, except that the fit effect of surprisal on mean/variance in reading time is forced to be linear.

**Figure C1.**
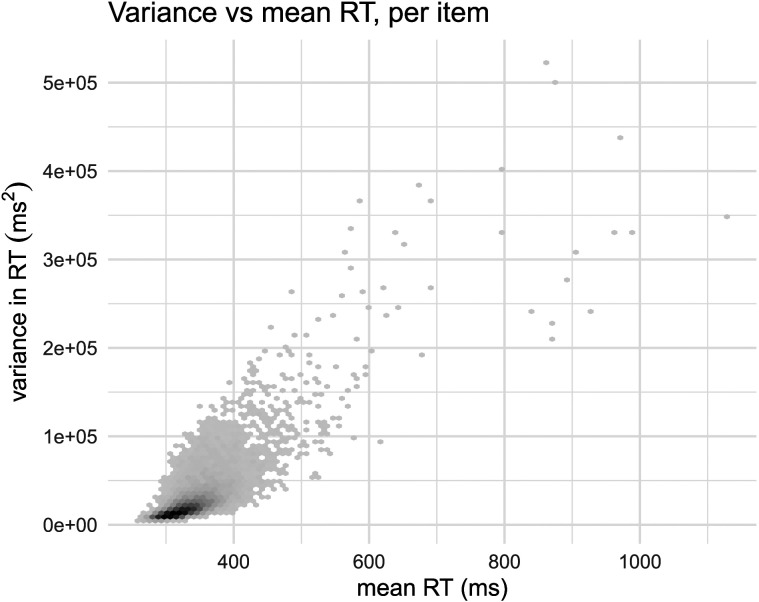
**Variance in self-paced reading time versus mean, by item in the Natural Stories corpus**. Variance increases with mean.

**Figure F1.**
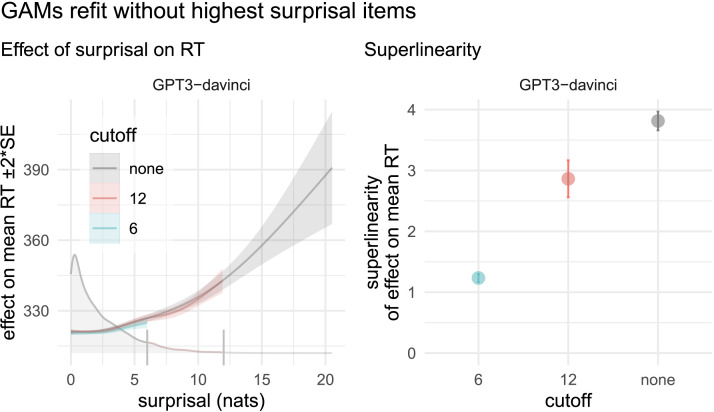
**GAMs fit on GPT-3 Davinci surprisals with highest surprisal items removed.** **Left**: The effect of surprisal on mean RT, fit on data subset with surprisal ≤ 6 (blue) and ≤ 12 (red). For comparison we also plot the fit on all data (grey; repeated from Figure 2). **Right**: Superlinearity of these curves (grey point repeated from Figure 4).

**Formula 3.**
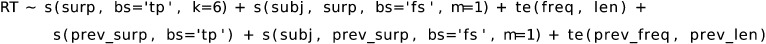
The mgcv formula for the nonlinear GAM with constant variance.

**Formula 4.**
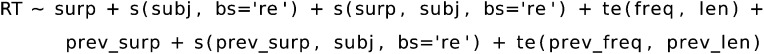
The mgcv formula for the linear control GAM with constant variance. The interpretation of this formula is essentially the same as that of formula 3, except that the effect of surprisal on reading time is assumed to be linear.

**Figure G1.**
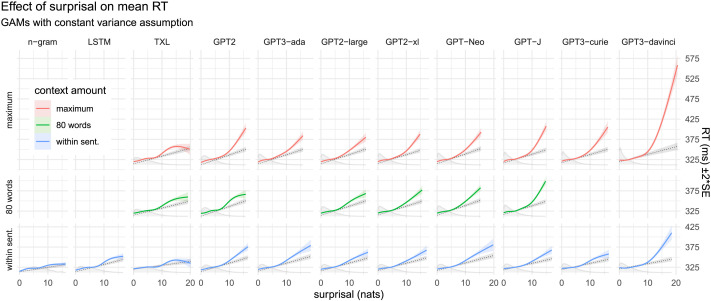
**The effect of surprisal on self-paced reading time from GAM models which assume constant variance (formulae 3 and 4).** Solid lines are the fitted effects from the nonlinear GAMs, dashed lines beneath are from the corresponding linear control GAMs. Shaded regions represent 95% CIs. Cf. Figure B1, top panel (effect on mean RT).

**Figure G2.**
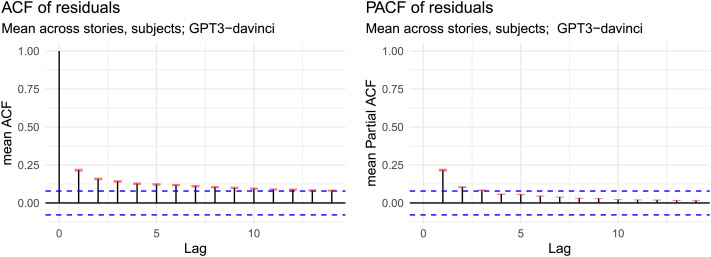
**Plots of mean autocorrelation function (ACF; left) and mean partial autocorrelation function (PACF; right) of residuals for the nonlinear GAM for GPT-3 Davinci.** For a given lag value, bar height represents the mean (P)ACF across stories and subjects, with 95% CI in red. Dashed blue lines indicate significance thresholds (against white noise null hypothesis).

**Figure G3.**
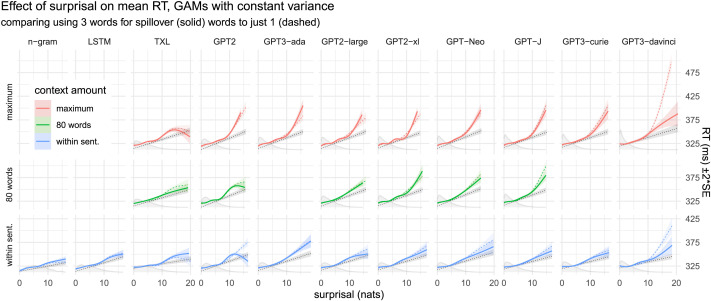
**Comparing the relationship between surprisal and RT using GAMs with spillover control predictors for three previous words (solid lines) to GAMs with only one word for spillover (dashed lines, repeated from Figure G1).** Linear control models plotted as dotted lines (also repeated from Figure G1). All GAMs for this plot were fit with an assumption of constant variance.

**Formula 5.**
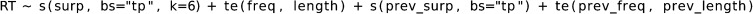
The mgcv formula for nonlinear GAM fits without by-subject effects. Mean reading time is predicted as a nonlinear global effect of surprisal, controlling for interactions between log frequency and orthographic length, all for the current word as well as the previous. Compare to formula 3, which also includes factor smooths by subject.

**Figure G4.**
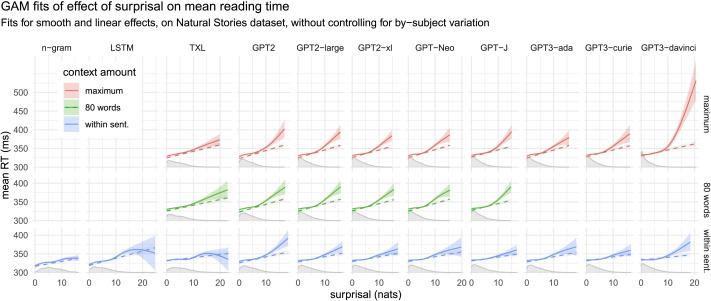
Plots of effect of surprisal on mean RT for constant-variance GAMs which do not control for by-subject differences (formula 5).

**Figure G5.**
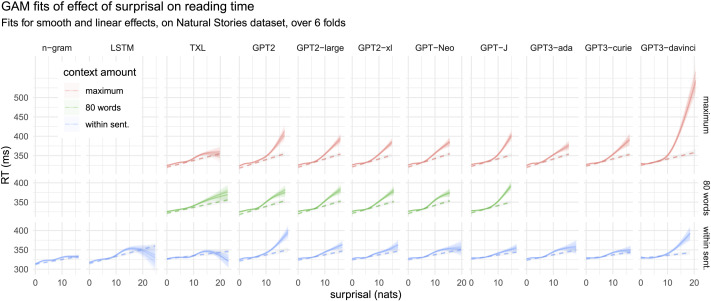
**Plots as in Figure G1, except that here we plot fitted curves for each of 6 GAMs fit on randomized folds of 5/6ths of the dataset.** Similarity across folds to suggests the models are not overfitting.

## APPENDIX A

### Runtime Variance of Guessing Without Replacement

In Guessing Without Replacement Algorithm in the main text we gave an expression for Var[*N*], the variance in the number of draws needed in guessing without replacement (eq. 7). Here we give the derivation of that expression. From general identities about covariance, we have the following.

$$\begin{array}{l} \text{Var}\left[N\right]=\text{Var}\left[N-1\right]=\text{Var}\left[\sum_{i}X_{i}\right]=\sum_{i,j}\text{Cov}\left[X_{i},X_{j}\right]\\ \;=\sum_{i,j}𝔼\left[X_{i}X_{j}\right]-𝔼\left[X_{i}\right]𝔼\left[X_{j}\right] \end{array}$$

In each element of this sum, the first expectation term 𝔼[*X*_*i*_*X*_*j*_] is simply the probability that items *i* and *j* both come before the target, 0. There are two cases to consider. If *i* = *j* this simplifies to 𝔼[$X_{i}^{2}$] = 𝔼[*X*_*i*_] = Pr(*i* ≺ 0) = $\frac{u_{i}}{u_{i}+u_{0}}$. Otherwise *i* ≠ *j*, and we have

$$\begin{array}{l} 𝔼\left[X_{i}X_{j}\right]=\mathrm{Pr}\left(i≺0,j≺0\right)\\ \;=\mathrm{Pr}\left(i≺j≺0\right)+\mathrm{Pr}\left(j≺i≺0\right) \end{array}$$ (A1)

where, by an argument similar to that given in the proof of proposition 1,

$$\begin{array}{l} \text{Pr}(i≺j≺0)=\text{Pr}(i≺j\wedge j≺0)=\text{Pr}(i≺j|j≺0)\text{Pr}(j≺0)\\ \;=\text{Pr}(i≺(j\vee 0))\text{Pr}(j≺0)\\ \;=\frac{u_{i}}{u_{i}+u_{j}+u_{0}}\frac{u_{j}}{u_{j}+u_{0}} \end{array}$$ (A2)

and likewise for Pr(*j* ≺ *i* ≺ 0).

So,

$$\begin{array}{l} \text{Var}[N]=\underset{i,j}{\sum }𝔼[X_{i}X_{j}]-𝔼[X_{i}]𝔼[X_{j}]\\ \;=\underset{i}{\sum }𝔼[X_{i}]-{\left(𝔼[X_{i}]\right)}^{2}+\underset{i\neq j}{\sum }𝔼[X_{i}X_{j}]-𝔼[X_{i}]𝔼[X_{j}]\\ \;=\underset{i}{\sum }\left[\frac{u_{i}}{u_{i}+u_{0}}-{\left(\frac{u_{i}}{u_{i}+u_{0}}\right)}^{2}\right]+\underset{i\neq j}{\sum }\left[\left(\frac{u_{i}}{u_{i}+u_{j}+u_{0}}\frac{u_{j}}{u_{j}+u_{0}}+\frac{u_{j}}{u_{i}+u_{j}+u_{0}}\frac{u_{i}}{u_{i}+u_{0}}\right)-\frac{u_{i}}{u_{i}+u_{0}}\frac{u_{j}}{u_{j}+u_{0}}\right] \end{array}$$ (A3)

This is the expression for variance given in eq. 7, and plotted in Figure 1 for Pareto-distributed weights.

## APPENDIX B

### Language Model Surprisals

For our surprisal estimates, we used the pretrained models from Huggingface Transformers (Wolf et al., 2020) identified by the following model IDs: transfo-xl-wt103, gpt2, gpt2-large, gpt2-xl, EleutherAI/gpt-neo-2.7B, and EleutherAI/gpt-j-6B. For the proprietary GPT-3 models, we used log probabilities provided via the OpenAI API for the original GPT-3 base models with model IDs davinci, curie, and ada the (accessed with free trial account, March, 2022). For the *n*-gram and LSTM models, as well as unigram frequency predictors, we use data made available in Boyce (2020, 2022). Code we used for retrieving all surprisal estimates we use will be released with supplemental material.

For each of the Transformer-based LMs, we obtain surprisal estimates with different amounts of context: In addition to the *maximum* context and *within-sentence* context amounts described in the main text, we also computed surprisals using *80 words* of context for the Huggingface models. These surprisals were estimated for each token using a sliding window of at most 80 tokens immediately preceding it within the story.

Figure B1 is the full version of Figure 2, giving the GAM fits for the overall effect of surprisal on reading time, for surprisals estimated by each of language models we use and each of the context amounts.

### Tokenization

Because of tokenization differences between the reading time corpus and the language models, some words seen by participants as single units correspond to multiple tokens according to the tokenizers used by the language models. In order to avoid making unnecessary assumptions, we discard words where the tokenization is different (excluding punctuation and whitespace differences). Because the different language models use different tokenization schemes, the set of corpus tokens we use differs across language models, though not substantially.^33^

We do not estimate surprisal for the first word in each text (or sentence, for LMs using only within-sentence context), and so these words are removed before fitting the models. Similarly, words immediately following an excluded word are also excluded since their previous-word surprisal predictor (included to control for spillover) is undefined.

### Comparison of LM surprisals

Figure B2 gives comparison of selected language models’ surprisals against each other, by item in the corpus. We can see that as the language models get lower mean surprisal, not all words’ surprisal is lowered proportionally. Also, as is clear from the density plots, at the higher end of surprisal, there is very little data, especially for the better language models. Given that it is in the high surprisal region that the predicted reading times according to the nonlinear GAMs we fit differ most from the predictions of the linear control, it is crucial to have data with constructions with high surprisal, something which is increasingly difficult with lower-perplexity language models.

## APPENDIX C

### Generalized Additive Models

Generalized additive models (GAMs; Wood, 2004, 2017) are a family of statistical models which allow nonlinear functions to be captured as linear combinations of basis functions. GAMs are a nonlinear generalization of generalized linear models, and as such similarly allow the use of different response distributions and linking functions. For our purposes, a GAM allows us to fit a regression of the form

$$\text{Time}\left(w_{n}\right)=f_{\theta }\left(I\left(w_{n}\right)\right)$$ (C1)

where the function *f*_*θ*_ is the linking function (as in previous literature since Smith & Levy, 2008a). GAMs are fit using penalized regression, of the form,

$$\underset{\theta }{\mathrm{argmax}}\left\{\text{likelihood}\left(f_{\theta }\right)-\lambda J\left(f_{\theta }\right)\right\}$$ (C2)

where the ‘wiggliness’ penalty functional *J* is specified so that *J*(*f*_*θ*_) = 0 if *f*_*θ*_ is linear, and, crucially, wiggliness is controlled by a parameter *λ*, which controls the trade-off between smoothness and fit to the data. This parameter itself may fit by cross validation, so the resulting regression model will be only be as nonlinear as necessary.

For our GAMs, we use the implementation provided by gam in mgcv 1.8-40 using R 4.2.1 (R Core Team, 2021; Wood, 2017). All GAMs we report in the main text were fit using with the default restricted maximum likelihood (REML) method for smoothing parameter estimation. Additional models given in this appendix that have a constant-variance assumption were fit using the more efficient mgcv::bam routine, and fast REML (fREML) for smoothing parameter estimation for computational efficiency.

### Nonlinear GAM Details

Formula 1 gives the mgcv formulae we use for the GAM fits of the nonlinear effect of surprisal on reading time. We fit Gaussian location-scale models (Rigby & Stasinopoulos, 2005; Wood et al., 2016), which lets us specify smooth predictors for the mean and standard deviation separately (with family=gaulss()). The LHS of the first formula specifies the structure of the linear predictor for mean RT, and the second that for standard deviation. In all our models, we use the default links: identity link for the mean, and log link for the standard deviation (so the relationship between the linear predictor and the standard deviation is *η* = log(*σ* + *b*), with parameter *b* = 0.01).

For the predictors of mean, following Smith and Levy (2013), Goodkind and Bicknell (2018), and Wilcox et al. (2020), we include a nonlinear term for the main effect of surprisal, and also include a tensor product term for the interaction between log-frequency and word length (orthographic) of the current word. Also following this previous literature, we include predictors likewise for the effect of the previous word on current reading time, to help control for spillover effect (see discussion in, e.g., Smith & Levy, 2013). We additionally include subject-specific terms (using bs='fs' in mgcv to use the factor-smooth interaction basis) to allow for by-subject nonlinear effects on reading time, to avoid the assumption of linearity, rather than just random slopes and or intercepts as in Goodkind and Bicknell (2018). Unlike by-subject random smooths, which fit potentially nonlinear effects independently for each subject (or separate by-subject models, as used by Smith & Levy, 2013, for their experiment with eye-tracking data only), including the subject predictor as a factor-smooth interaction allows us to control for potentially different nonlinear effects of each participant (and random intercept) while sharing the same smoothing parameter, as is appropriate for by-subject random smooths (Wood, 2017, §7.7.4).^34^

#### Basis and order of penalty term.

Since we are particularly interested in the shape of this curve in the high-surprisal region, where there is the least data, we choose not to use cubic regression splines (unlike Goodkind & Bicknell, 2018; Wilcox et al., 2020), for which knot locations are by default chosen by quantile. Instead we use thin-plate regression splines (TPRS; Wood, 2003), avoiding the problem of knot placement. Using TPRS results in evenly distributed knots.

We set the order of the penalty functional to 1 (m=1) in the factor smooth, which penalizes towards a slope of zero (flat line). This results in penalizing deviation from the global effect, limiting the wiggliness per speaker, suitable for these by-speaker nonlinear effects (cf. Wieling et al., 2016). While this choice is principled, changing it does not affect our qualitative results. Our choice to set the penalty term m=1 in the factor smooth interaction terms is motivated by the fact that the default m=2 would allow more wiggliness per speaker smooths, and could lower our power to detect the population-level positive effect. In preliminary testing with m=2, the qualitative results were unchanged. We note however that the confidence intervals on the resulting main smooths were somewhat wider than the results using m=1 which we report, and to this extent, the choice may be somewhat anticonservative. Since we are interested in the overall effect, the choice to set a stronger penalty on the factor smooths is warranted, and follows previous literature on using similar GAMs (e.g., Sóskuthy, 2021; Wieling et al., 2016), though we are the first to introduce it to this application.

#### Restricting maximum wiggliness.

We must choose a value for basis dimension for the main smooth term, *k*. This parameter effectively controls the maximum degrees of freedom of the curve, with a higher values allowing a potentially very wiggly curve to be fit, while at the lower value, the curve would be forced toward the null space (linear). The arbitrary default in mgcv is *k* = 10. Some previous work chooses a large number for the basis dimension (such as *k* = 20, in e.g., Smith & Levy, 2013; Wilcox et al., 2020) and allows the smoothing parameter to be fit according to the data, resulting in only as smooth a curve as is necessary. Instead, we set *k* = 6, effectively allowing a maximum of 5 degrees of freedom (*k* − 1, because one degree is lost to the identifiability constraint). The result is nonlinear effects which are restricted to simpler curves. We limit the basis dimension since we are in particular interested in the rather simple question: given a few degrees of freedom, whether the GAM will use them to bend the curve, or not. In preliminary experimentation, increasing the basis dimension leads to local nonlinearities which obscure the global pattern somewhat, but don’t change the qualitative interpretation.

### Linear Control GAM Details

As described in the main text (Generalized Additive Models), for each language model and context amount, in addition to the GAM fit using formula 1 (the nonlinear GAM), we also fit a GAM using formula 2 (the linear control GAM), where the effects of surprisal on reading time mean and likewise on variance are assumed to be linear, but otherwise the model is the same. For this linear control, the global nonlinear terms of surprisal and previous word surprisal are replaced with linear parametric terms, and the factor-smooth subject terms are replaced with linear random effects (via the basis bs='re'). One caveat is that this model specification includes the additional assumption that the random slopes and intercepts are independent, which is not assumed in the case of the nonlinear model.^35^ We leave the tensor product terms for the interactions between frequency and length the same for maximum similarity between the two. The interpretation of the linear control models is as a baseline to which the nonlinear models would converge if the true effect of surprisal on reading time were perfectly linear.

### Significance of Superlinearity

We are interested in whether an assumption of linearity is justified to model the effect of surprisal on processing difficulty, or if a nonlinear fit is necessary. One way to specifically test whether a smooth term may be replaced with a linear parametric term in a GAM is to explicitly separate the basis for the penalty range space from the basis for the null space when parametrizing the smooth, effectively allowing one to ask the question “is this curve significantly nonlinear?” This technique can be accomplished in mgcv with thin-plate regression splines by setting the smooth up without a null space basis, and including a parametric term (as described in Wood, 2017, §6.12.3).^36^ For our purposes, we are interested in the shape of the nonlinear fit (namely, whether it is superlinear), not simply whether it is significant. Nonetheless, we experimented with using this technique to get a *p*-value testing whether the nonlinear components were required. Unsurprisingly, we found across models that the nonlinear components were significant, though not in an illuminating way: even for the worst LMs and the most qualitatively linear fits, there are small but statistically significant nonlinearities. For this reason this technique is not a useful way to quantify nonlinearity.

### Nonconstant Variance of Data

Most modelling of the relationship between surprisal and reading time, both using generalized linear mixed models and GAMs, has used the default Gaussian distribution for the dependent variable, with identity linking function. The primay exceptions are Hofmann et al. (2022), who use a gamma family with the default logarithmic linking function, and Smith and Levy (2013), who also mention that their results were robust to switching from Gaussian to a heavy-tailed (gamma) family. The choice of dependent variable distribution and linking function for models of reading time data in general is explored in detail in Lo and Andrews (2015), who point out that RTs are better modelled by waiting time distributions such as the gamma or inverse-Gaussian.

Looking at the reading time data we use empirically, before fitting any models, it is clear that the variance in reading time is not constant across mean reading time values, as illustrated in Figure C1. This already suggests that the assumption of constant error variance implicit in using least squares estimation (constant Gaussian distributed error) is not warranted. This lack of constant variance is a known feature of reaction time data, and motivates the use of a response distribution that is better matched to these data (see Lo & Andrews, 2015, for detailed discussion). In fitting Gaussian scale-location models (Wood et al., 2016) where variance is allowed to vary as a smooth function of the predictors, we can effectively probe the correspondance between mean and variance. In our results, the similarity between the fitted curves for mean and variance (Figures 2 and B1) suggest that use of a member of the exponential family for which variance increases smoothly with the predictor value is indeed justified (for example, gamma or inverse-Gaussian distributions). An expansion of the current study using such models is material for future work.

### Relationship between Mean and Variance

The GAMs we fit did not assume any particular relationship between RT and variance in RT. Yet, comparing the nonlinear GAM’s mean and variance fits for a given LM in Figure 2, it is clear that these two curves are generally similar to each other in shape. The similarity between these fitted curves may justify the use of statistical models where variance is a *assumed* to be a fixed increasing function of the predicted mean.^37^ Making this assumption *a priori*, rather than fitting that relationship simultaneously for mean and variance, as we did, would have the benefit of making the models much less computationally costly to fit. We leave to future work the exploration of models with variance that scales parametrically with the mean.

## APPENDIX D

### Comparison with Shain et al. (2022)

Shain et al. (2022) present a meticulous and large-scale study of the relationship between surprisal and processing difficulty, using multiple datasets (including Natural Stories) and reading modalities (eye-tracking and Maze task data, in addition to self-paced reading) and using surprisal estimates from multiple language models (including a 5-gram model, and GPT-2, GPT-J, and GPT-3 Davinci as well as a PCFG model and cloze probabilities). Unlike the current study and much previous literature, Shain et al. (2022) do not use GAMs, but instead make use of continuous-time deconvolutional regressive neural networks (CDRNNs; Shain & Schuler, 2021, 2022), a new modelling technique which describes the influence of predictors in terms of overlapping additive impulse response functions in continuous time. This technique also allows modelling of the effect of predictors on all parameters of the response distribution (not just the mean), with full nonlinear random effects.

While their study and the empirical component of our study both target the shape of the linking function, and use surprisal estimates from some of the same pre-trained language models, the differing analytical models make it difficult to compare results directly. Still, for the Natural Stories dataset (which, of the datasets they include, has the largest number of observations, and is also the dataset we use), they report qualitative confirmation of the superlinear relationship we observe between surprisal and self-paced reading time. Namely, their results for this data show curves that increase superlinearly with surprisal for the larger LMs, with superlinear models tending also to show stronger performance (larger psychometric predictive power). However, they do not find such a trend in the other datasets and modalities, and find that *overall* (when aggregating across all and datasets and modalities) the larger models GPT-3 and GPT-J perform worse as psychometric models than GPT-2, especially if the linking function is constrained to be linear^38^. Their overall conclusion is that empirical evidence favors a linear relationship.

As discussed in the main text, we believe our choice of the Natural Stories dataset is well-motivated, given the design of the corpus, a well as the large number of participants, which allows us to better control for a large amount of potential variation between individuals. However, the difference between the results on this dataset, which do show superlinearity (in both our study and theirs), and those on the other datasets and modalities in their study, which do not, complicates the picture. It is also worth noting (as Shain et al., 2022 do) that if the uncertainty interval covers an a superlinear function, it is not possible to falsify the hypothesis of superlinearity in favor of a linear linking function. This observation leads back to our fundamental motivating question: What predictions do algorithmic theories of processing make about the relationship between surprisal and processing difficulty? In this work we have argued that the only algorithms we know of which naturally scale in surprisal predict a superlinear linking function. The tension between this prediction and the results of Shain et al. (2022) motivate further study from both empirical and theoretical directions.

## APPENDIX E

### Surprisal Explorer

To facilitate exploration of the words of the corpus in full context, with language model surprisal estimates and reading time annotations, we provide an interactive utility available at github.com/mcqll/plausibility-sampling-processing/.

## APPENDIX F

### Effect of Highest Surprisal Words

The difference between a linear and superlinear linking function is naturally most appreciable in the high end of the surprisal range. However, for low-perplexity LMs, the vast majority of words in the corpus are relatively low surprisal, as can be seen in the highly skewed density plots of surprisal values (plotted above in Figures 2 and B1, and compared across LMs in Figure B2). This is to be expected for any corpus of fluent text, and remains true of the Natural Stories corpus, despite its being designed to contain rare and marked constructions. Since this skew is especially pronounced for the lowest-perplexity LMs, the models for which we see the most superlinearity are also the models for which we have the smallest amount of data in the high end of the surprisal range. To understand how the particular words in this region of the surprisal range affect our results, in this appendix we take a detailed look at the highest-surprisal words according to GPT-3 Davinci—the lowest-perplexity of the LMs we use, and the one for which the relationship with reading time is the most superlinear. Then we assess their contribution to this superlinearity, by re-fitting the GAM without these words.

### Highest Surprisal Words

For GPT-3 Davinci, the top 40% of the surprisal range (surprisal > 12 nats) contains only about 0.3% of the words in the corpus. Table F1 gives each of these words, in order of decreasing surprisal, with part-of-speech tag and dependency label (provided with the Natural Stories corpus; see Futrell et al., 2021, §2.3).

**Table F1.** All 19 words in the corpus with GPT-3 Davinci surprisal >12, with surrounding context, mean RT, part of speech tag, and dependency label annotations from the parses provided with the corpus.

word in context		GPT-3 D.surprisal	POS	dependencylabel	story #	word #	meanRT
1	… in military programs **US** conducted in the …	20.51	NNP	nsubj	8	836	413.83
2	… mania in February **sixteen** thirty-seven, tulip …	18.11	NN	compound	9	38	862.11
3	… His brother had **blatantly** peeked and even …	15.50	RB	advmod	2	748	391.25
4	… movie brought the **nineteen** forty-seven incident …	14.73	CD	nummod	8	404	361.44
5	… names, such as **even** ‘Admiral of Admirals’ and …	14.49	RB	advmod	9	343	460.22
6	… classic that many **publishing** houses continue …	14.22	NN	compound	9	884	317.26
7	… well which seems **puzzling** at first, but the reason …	13.84	JJ	xcomp	1	137	375.21
8	… the little bird **guarded** by the owl peeped out, …	13.62	VBN	acl	4	904	326.08
9	… who Abby still **strained** to remain upset with, …	13.23	VBN	dep	6	772	374.57
10	… sight, and then **folding** his wings together, he …	13.12	VBG	dep	4	479	359.07
11	… was called and **though** they understood the birds …	13.10	IN	mark	4	37	366.11
12	…were supposed to **slowly** wait to be called, I …	12.72	RB	advmod	5	448	346.46
13	… little girl no one **sheltered** from the gelid air …	12.59	VBD	acl:relcl	3	28	439.62
14	… markets, which **merchants** used to sell and buy …	12.38	NNS	nsubj	9	544	330.08
15	… vocalizations, which **motor** tics typically precede, …	12.27	NN	compound	10	315	389.39
16	… September, and thus **actual** purchases occurred …	12.13	JJ	amod	9	488	388.97
17	… the boar? By the **handsome** reward many felt …	12.10	JJ	amod	1	346	355.48
18	… who they knew looked **dirt** poor and helpless …	12.04	RB	advmod	3	978	368.29
19	… The Dutch Golden Age **growers** named their …	12.02	NNS	nsubj	9	297	387.70

Inspecting each of the words on this list in context, it is possible to identify intuitive reasons why it is plausible that they would be high-surprisal for humans, yet it is not possible to put them into one common category. Most are examples of unusual grammatical constructions. The notable exceptions are items 1, 2, and 4: The highest surprisal word (item 1) seems to be the result of a typo or at least unconventional usage (“**US**” rather than “the US”). Also high on the list are two numbers which are dates written out longform (items 2 and 4 in the table), where presumably numerals would be more expected. Of the remaining items on the list, many are examples of the kinds of marked syntactic constructions that Natural Stories is designed to contain. For example, four are words at critical regions in object-extracted relative clauses (ORCs). Two are on the verb (item 13: “little girl [_CP_ ⌀ no one **sheltered** …]” and 9: “Mom, [_CP_ who Abby still **strained** to …]”), and the other two the onset of the subject NP (item 14: “markets, [_CP_ which **merchants** used …]”, and 15: “vocalizations [_CP_ which **motor** tics …]”).^39^ Item 7 is at the critical region of a garden path sentence: “It shows a sinister looking boar’s head sitting on top of a well [which seems **puzzling** at first]”—the word “**puzzling**” disambiguates attachment ambiguity for the relative clause, in favor of the matrix CP as the subject, rather than the local NP “well”. Item 19 is another where temporary ambiguity is resolved in favor of the less-likely alternative “The Dutch Golden Age **growers** …”, a noun following an NP modifier, where presumably a verb would be more expected. Item 8, “Then the little bird **guarded** by the owl peeped out, …” is in an example of main verb / reduced-relative (MV/RR) garden-path, however the surprising word comes *before* the disambiguating word in the noun phrase (where surprisal-based processing difficulty is theoretically predicted). Item 11 begins a CP subordinating conjunction “A meeting of all the birds was called and [**though** they understood the birds … would be unable to come], many birds came from faraway meadows and woods.” Item 10 is a gerund modifier “… and then **folding** his wings together, he sank to earth …”. The remaining handful of words are other somewhat rare modifiers (items 3, 5, 6, 12, 17, 18), which are plausibly hard to predict especially given they come before their heads. Note that for the purpose of understanding the empirical relationship between surprisal and processing time, what matters about these words is simply that they are surprising. It is reassuring to see that for the most part they seem like items which would be intuitively hard for humans to predict.

### Models without Highest Surprisal Words

To determine the extent to which our conclusions about superlinearity rely on the relatively few highest-surprisal items, we re-fit nonlinear GAMs (formula 1) including only those items in the corpus with surprisal below a cutoff value:

$$\left\{w\in \text{Corpus}:I\left(w\right)\leq I_{\text{cutoff}}\right\}.$$

We fit two versions of this control: one with I_cutoff_ = 12, and and one with I_cutoff_ = 6. Cutting off above surprisal threshold I_cutoff_ = 12 removed the 19 words discussed above in Table F1 (which comprise 1557 RT observations, roughly 0.3% of total observations in the data). Cutting off above I_cutoff_ = 6 removed an additional 470 words (489 words total, comprising 41261 RT observations, roughly 7.6% of total observations in the data).

Figure F1 (left) shows the fitted effect of surprisal on mean RT from these GAMs (I_cutoff_ = 12 in red, I_cutoff_ = 6 in blue), compared to the model fit on all words (grey, repeated from Figure 2). Figure F1 (right) shows the superlinearity of these curves. We observe that the exclusion of these high-surprisal items leaves the shape of the curve basically unchanged in the remaining lower-surprisal region. Truncating the curve like this naturally reduces the amount of superlinearity we see, but the curve remains superlinear, even with the more drastic cutoff.

## APPENDIX G

### Additional Controls

#### Gaussian GAMs with constant variance assumption.

For comparison with the GAMs discussed in the main text, which fit the effect of surprisal on variance in reading time as well as mean, we also fit versions of these models with a constant variance assumption (formulae 3 and 4). In addition to allowing a more direct comparison with previous work, which has largely used Gaussian constant-variance GAMs (Goodkind & Bicknell, 2018; Hofmann et al., 2022; Smith & Levy, 2008a, 2013; Wilcox et al., 2020), these models also function as a control for the effect that fitting variance might have had on the shape of the relationship with mean RT. They also have the benefit of being much less costly to compute than the models which must fit the effect on variance as well as mean of the response.

Figure G1 shows the relationship between surprisal and RT according to these models (compare with the mean effect in Figure B1). As with the results presented in the main text, these results show increasing superlinearity with LM quality.

### Spillover and Autocorrelation

When fitting a mixed-effects model or GAM to predict reaction time data, it is common practice to include additional predictors for the previous word—or, more generally all words within a *M*-word window including the current word to control for spillover effects (D. C. Mitchell, 1984; Vasishth, 2006). For our models, we follow previous literature in this area (e.g., Goodkind & Bicknell, 2018, 2021; Meister et al., 2021) in including predictors for one previous word for spillover control (*M* = 2). However, some other studies (e.g., Wilcox et al., 2020) have used *M* = 4, following Smith and Levy (2013) who noted that a window size of *M* = 4 was empirically best to capture the effect of surprisal on self-paced reading time in their study. For our models, we found that including more than one previous word was computationally intractable, since predictors for each additional spillover word adds a full set of by-subject nonlinear effects for both location and scale.^40^ In this section we investigate the degree to which this choice could have affected our results.

#### Autocorrelation plots.

One way to assess whether a larger *M* would have likely affected our results is to look for residual autocorrelation in our models. Intuitively, spillover effects cause time-dependence in the response, since higher surprisal on a word will result not just in higher reading time on the current word, but this effect will also “spill over” to the subsequent word (or words). Intuitively, if such time-dependence is not fully captured by our models, this will result in time-dependence in the residuals. We can look for evidence of such time-dependence by looking for autocorrelation in the residuals.

Figure G2 shows the mean (complete) autocorrelation (left) and mean partial autocorrelation (right) for the nonlinear GAM fit on GPT-3 Davinci surprisals, averaged across stories and subjects.^41^ 95% CIs are shaded red. Autocorrelation for GAMs fit on surprisals from other LMs are similar.

These plots indicate that there amount of residual autocorrelation is small for any lag. In the PACF plot, for all *k* > 3 partial autocorrelation is not significantly different from zero, and even for *k* ≤ 3, partial correlation values are small. This suggests that optimally we should include predictors for three previous words (*M* = 4), but we may expect that doing so would not have a large effect on results.

#### Additional predictors for spillover.

We also experimented with fitting the simpler constant-variance models (described above in the first subsection of this appendix), but with predictors for the previous three words, to control for spillover.

These GAMs are plotted in Figure G3 (solid lines), together with GAMs with only one previous word (dashed lines; repeated from Figure G1), for comparison. Grey dotted lines are the linear control models (also repeated from Figure G1). We observe that in most cases there is little difference between the curves with three spillover words compared to those with only one: Some fits become slightly more visually superlinear, and others slightly less. One large change is in GPT-3 Davinci, which does become much less steeply superlinear in the high end of the surprisal range, but remains superlinear overall.

### Without by-subject Effects

Unlike our study, Wilcox et al. (2020) use GAMs to model mean item reading time as the response, and do not control for by-subject random effects. For comparison with their results, we also fit models of mean RT without the by-subject effects (formula 5). These models were fit with a constant-variance assumption, for computational efficiency, given that the superlinearity we observed was robust to this simplifying assumption, as discussed above. Figure G4 (analogous to Figure B1) provides plots of GAMs fit with this formula. The results show much larger confidence intervals, suggesting that properly modelling by-subject variation in this data gives us higher power to detect population-level nonlinear effects.

### GAM Plots from Folds of Data

To insure against potential high-leverage outliers, we carried out a cross-validation control. For this control, we partitioned the data into 6 folds, and refit the GAMs 6 times leaving out one fold each time. These models were fit with a constant-variance assumption, for computational efficiency (as with the previous control).

The fitted effect of surprisal on reading time for each of the 6 folds, with confidence intervals, are plotted superimposed in Figure G5. Comparing these results with the plots for GAMs fit on all of the data in Figure B1 we can visually confirm that the results are effectively identical, and conclude that the superlinearity we see is robust.

## References

[bib1] Abney, S. P., & Johnson, M. (1991). Memory requirements and local ambiguities of parsing strategies. Journal of Psycholinguistic Research, 20(3), 233–250. 10.1007/bf01067217

[bib2] Agapiou, S., Papaspiliopoulos, O., Sanz-Alonso, D., & Stuart, A. M. (2017). Importance sampling: Intrinsic dimension and computational cost. Statistical Science, 32(3), 405–431. 10.1214/17-STS611

[bib3] Anderson, J. R. (1990). The adaptive character of thought. Psychology Press. 10.4324/9780203771730

[bib4] Anderson, J. R. (1991). Is human cognition adaptive? Behavioral and Brain Sciences, 14(3), 471–485. 10.1017/S0140525X00070801

[bib5] Anderson, J. R., & Lebiere, C. (1998). The atomic components of thought. New York: Psychology Press. 10.4324/9781315805696

[bib6] Arehalli, S., Dillon, B., & Linzen, T. (2022). Syntactic surprisal from neural models predicts, but underestimates, human processing difficulty from syntactic ambiguities. In Proceedings of the 26th Conference on Computational Natural Language Learning (CoNLL) (pp. 301–313). https://aclanthology.org/2022.conll-1.20

[bib7] Aurnhammer, C., & Frank, S. L. (2019). Comparing gated and simple recurrent neural network architectures as models of human sentence processing. In Proceedings of the 41st Annual Conference of the Cognitive Science Society (pp. 112–118). https://hdl.handle.net/2066/213724

[bib8] Balota, D. A., Pollatsek, A., & Rayner, K. (1985). The interaction of contextual constraints and parafoveal visual information in reading. Cognitive Psychology, 17(3), 364–390. 10.1016/0010-0285(85)90013-1, 4053565

[bib9] Berwick, R. C., & Weinberg, A. S. (1982). Parsing efficiency, computational complexity, and the evaluation of grammatical theories. Linguistic Inquiry, 13(2), 165–191. https://www.jstor.org/stable/4178272

[bib10] Bicknell, K., & Levy, R. (2010). A rational model of eye movement control in reading. In Proceedings of the 48th Annual Meeting of the Association for Computational Linguistics (pp. 1168–1178). https://www.aclweb.org/anthology/P10-1119

[bib11] Bicknell, K., & Levy, R. (2012). Word predictability and frequency effects in a rational model of reading. In Proceedings of the 34th Annual Meeting of the Cognitive Science Society (Vol. 34, pp. 126–131). https://cogsci.mindmodeling.org/2012/papers/0035/

[bib12] Black, S., Leo, G., Wang, P., Leahy, C., & Biderman, S. (2021). GPT-Neo: Large scale autoregressive language modeling with mesh-tensorflow. 10.5281/zenodo.5297715

[bib13] Boston, M. F., Hale, J., Kliegl, R., Patil, U., & Vasishth, S. (2008). Parsing costs as predictors of reading difficulty: An evaluation using the Potsdam Sentence Corpus. Journal of Eye Movement Research, 2(1). 10.16910/jemr.2.1.1

[bib14] Bouchard-Côté, A., Petrov, S., & Klein, D. (2009). Randomized pruning: Efficiently calculating expectations in large dynamic programs. In Y. Bengio, D. Schuurmans, J. Lafferty, C. Williams, & A. Culotta (Eds.), Advances in neural information processing systems (Vol. 22). Curran Associates, Inc. https://proceedings.neurips.cc/paper/2009/file/e515df0d202ae52fcebb14295743063b-Paper.pdf

[bib15] Boyce, V. (2022). Amaze-natural-stories. Retrieved September 24, 2022, from https://github.com/vboyce/amaze-natural-stories.

[bib16] Boyce, V., & Levy, R. (2020). A-maze of Natural Stories: Texts are comprehensible using the Maze task. In Talk at 26th Architectures and Mechanisms for Language Processing conference (AMLaP 26). Potsdam, Germany.

[bib17] Brothers, T., & Kuperberg, G. R. (2021). Word predictability effects are linear, not logarithmic: Implications for probabilistic models of sentence comprehension. Journal of Memory and Language, 116, 104174. 10.1016/j.jml.2020.104174, 33100508 PMC7584137

[bib18] Brown, T. B., Mann, B., Ryder, N., Subbiah, M., Kaplan, J., Dhariwal, P., Neelakantan, A., Shyam, P., Sastry, G., Askell, A., Agarwal, S., Herbert-Voss, A., Krueger, G., Henighan, T., Child, R., Ramesh, A., Ziegler, D. M., Wu, J., Winter, C., … Amodei, D. (2020). Language models are few-shot learners. In H. Larochelle, M. Ranzato, R. Hadsell, M.-F. Balcan, & H.-T. Lin (Eds.), Advances in Neural Information Processing Systems 33: Annual Conference on Neural Information Processing Systems 2020, NeurIPS 2020, December 6–12, 2020, Virtual. https://proceedings.neurips.cc/paper/2020/hash/1457c0d6bfcb4967418bfb8ac142f64a-Abstract.html

[bib19] Buys, J. (2018). Incremental Generative Models for Syntactic and Semantic Natural Language Processing. Retrieved June 14, 2022, from https://ora.ox.ac.uk/objects/uuid:a9a7b5cf-3bb1-4e08-b109-de06bf387d1d.

[bib20] Chatterjee, S., & Diaconis, P. (2018). The sample size required in importance sampling. Annals of Applied Probability, 28(2), 1099–1135. 10.1214/17-aap1326

[bib21] Chopin, N., & Papaspiliopoulos, O. (2020). An introduction to sequential Monte Carlo (1st ed.). Springer. 10.1007/978-3-030-47845-2

[bib22] Collins, M., & Roark, B. (2004). Incremental parsing with the perceptron algorithm. In Proceedings of the 42nd Annual Meeting of the Association for Computational Linguistics (pp. 111–118). 10.3115/1218955.1218970

[bib23] Costa, F. (2003). Towards incremental parsing of natural language using recursive neural networks. Applied Intelligence, 19(1/2), 9–25. 10.1023/a:1023860521975

[bib24] Dai, Z., Yang, Z., Yang, Y., Carbonell, J., Le, Q., & Salakhutdinov, R. (2019). Transformer-XL: Attentive language models beyond a fixed-length context. In Proceedings of the 57th Annual Meeting of the Association for Computational Linguistics (pp. 2978–2988). 10.18653/v1/p19-1285

[bib25] Demberg, V., & Keller, F. (2008). Data from eye-tracking corpora as evidence for theories of syntactic processing complexity. Cognition, 109(2), 193–210. 10.1016/j.cognition.2008.07.008, 18930455

[bib26] Dotlačil, J. (2021). Parsing as a cue-based retrieval model. Cognitive science, 45(8), e13020. 10.1111/cogs.13020, 34379334 PMC8459291

[bib27] Doucet, A., Freitas, N., & Gordon, N. (Eds.). (2001). Sequential Monte Carlo methods in practice. Springer. 10.1007/978-1-4757-3437-9

[bib28] Doucet, A., & Johansen, A. M. (2011). A tutorial on particle filtering and smoothing: Fifteen years later. In D. Crisan & B. Rozovskiǐ (Eds.), The Oxford Handbook of Nonlinear Filtering (pp. 656–704). Oxford University Press. https://www.stats.ox.ac.uk/~doucet/doucet_johansen_tutorialPF2011.pdf Note: Version 1.1 – December 2008 with typographical corrections March 2012.

[bib29] Earley, J. (1970). An efficient context-free parsing algorithm. Communications of the ACM, 13(2), 94–102. 10.1145/362007.362035

[bib30] Eberhard, K. M., Spivey-Knowlton, M. J., Sedivy, J. C., & Tanenhaus, M. K. (1995). Eye movements as a window into real-time spoken language comprehension in natural contexts. Journal of Psycholinguistic Research, 24(6), 409–436. 10.1007/BF02143160, 8531168

[bib31] Ehrlich, S. F., & Rayner, K. (1981). Contextual effects on word perception and eye movements during reading. Journal of Memory and Language, 20(6), 641. 10.1016/S0022-5371(81)90220-6

[bib32] Elvira, V., Míguez, J., & Djurić, P. M. (2017). Adapting the number of particles in sequential Monte Carlo methods through an online scheme for convergence assessment. IEEE Transactions on Signal Processing, 65(7), 1781–1794. 10.1109/TSP.2016.2637324

[bib33] Engelmann, F. (2016). Toward an integrated model of sentence processing in reading [Doctoral dissertation, Universität Potsdam]. Potsdam, Germany. Retrieved October 12, 2022, from https://publishup.uni-potsdam.de/frontdoor/index/index/docId/10086.

[bib34] Engelmann, F., Jäger, L. A., & Vasishth, S. (2019). The effect of prominence and cue association on retrieval processes: A computational account. Cognitive Science, 43(12), e12800. 10.1111/cogs.12800, 31858626

[bib35] Farmer, T. A., Misyak, J. B., & Christiansen, M. H. (2012). Individual differences in sentence processing. In K. McRae, M. Joanisse, & M. Spivey (Eds.), The Cambridge Handbook of Psycholinguistics (pp. 353–364). Cambridge University Press. 10.1017/CBO9781139029377.018

[bib36] Fernandez Monsalve, I., Frank, S. L., & Vigliocco, G. (2012). Lexical surprisal as a general predictor of reading time. In Proceedings of the 13th Conference of the European Chapter of the Association for Computational Linguistics (pp. 398–408). https://aclanthology.org/E12-1041

[bib37] Floridi, L., & Chiriatti, M. (2020). GPT-3: Its nature, scope, limits, and consequences. Minds and Machines, 30(4), 681–694. 10.1007/s11023-020-09548-1

[bib38] Fossum, V., & Levy, R. (2012). Sequential vs. Hierarchical syntactic models of human incremental sentence processing. In Proceedings of the 3rd Workshop on Cognitive Modeling and Computational Linguistics (CMCL 2012) (pp. 61–69). https://aclanthology.org/W12-1706

[bib39] Fox, D. (2003). Adapting the sample size in particle filters through KLD-Sampling. The International Journal of Robotics Research, 22(12), 985–1003. 10.1177/0278364903022012001

[bib40] Frank, S. L. (2009). Surprisal-based comparison between a symbolic and a connectionist model of sentence processing. In Proceedings of the 31st Annual Meeting of the Cognitive Science Society. Retrieved October 12, 2022, from https://escholarship.org/uc/item/02v5m1hf.

[bib41] Frank, S. L., Otten, L. J., Galli, G., & Vigliocco, G. (2013). Word surprisal predicts N400 amplitude during reading. In Proceedings of the 51st Annual Meeting of the Association for Computational Linguistics (Volume 2: Short Papers) (pp. 878–883). https://www.aclweb.org/anthology/P13-2152

[bib42] Frazier, L. (1987). Syntactic processing: Evidence from Dutch. Natural Language & Linguistic Theory, 5(4), 519–559. 10.1007/BF00138988

[bib43] Frazier, L., & Fodor, J. D. (1978). The sausage machine: A new two-stage parsing model. Cognition, 6(4), 291–325. 10.1016/0010-0277(78)90002-1

[bib44] Freer, C., Mansinghka, V. K., & Roy, D. (2010). When are probabilistic programs probably computationally tractable? In NIPS Workshop on Monte Carlo Methods for Modern Applications. https://montecarlo.wdfiles.com/local–files/contributed-abstracts/nipsmc2010_freer_etal.pdf

[bib45] Futrell, R. (2017). Memory and locality in natural language. https://hdl.handle.net/1721.1/114075

[bib46] Futrell, R., Gibson, E., & Levy, R. (2020). Lossy-context surprisal: An information-theoretic model of memory effects in sentence processing. Cognitive Science, 44(3), e12814. 10.1111/cogs.12814, 32100918 PMC7065005

[bib47] Futrell, R., Gibson, E., Tily, H. J., Blank, I., Vishnevetsky, A., Piantadosi, S. T., & Fedorenko, E. (2021). The Natural Stories corpus: A reading-time corpus of English texts containing rare syntactic constructions. Language Resources and Evaluation, 55(1), 63–77. 10.1007/s10579-020-09503-7, 34720781 PMC8549930

[bib48] Gao, L. (2021). On the sizes of OpenAI API models. EleutherAI Blog. Retrieved December 13, 2021, from https://blog.eleuther.ai/gpt3-model-sizes/.

[bib49] Gibson, E. (1998). Linguistic complexity: Locality of syntactic dependencies. Cognition, 68(1), 1–76. 10.1016/S0010-0277(98)00034-1, 9775516

[bib50] Gibson, E. (2000). The dependency locality theory: A distance-based theory of linguistic complexity. In A. Marantz, Y. Miyashita, & W. O’Neil (Eds.), Image, language, brain: Papers from the first mind articulation project symposium (pp. 94–126). Cambridge, MA: MIT Press. https://citeseerx.ist.psu.edu/viewdoc/summary?doi=10.1.1.592.5833&rank=1&q=The%20dependency%20locality%20theory:%20A%20distance-based%20theory%20of%20linguistic%20complexity.&osm=&ossid=

[bib51] Goodkind, A., & Bicknell, K. (2018). Predictive power of word surprisal for reading times is a linear function of language model quality. In Proceedings of the 8th Workshop on Cognitive Modeling and Computational Linguistics (CMCL 2018). 10.18653/v1/w18-0102

[bib52] Goodkind, A., & Bicknell, K. (2021). Local word statistics affect reading times independently of surprisal. 10.48550/ARXIV.2103.04469

[bib53] Graf, T., Monette, J., & Zhang, C. (2017). Relative clauses as a benchmark for minimalist parsing. Journal of Language Modelling, 5(1), 57–106. 10.15398/jlm.v5i1.157

[bib54] Gulordava, K., Bojanowski, P., Grave, E., Linzen, T., & Baroni, M. (2018). Colorless green recurrent networks dream hierarchically. In Proceedings of the 2018 Conference of the North American Chapter of the Association for Computational Linguistics: Human Language Technologies, (Volume 1: Long Papers) (pp. 1195–1205). 10.18653/v1/N18-1108

[bib55] Hale, J. T. (2001). A probabilistic Earley parser as a psycholinguistic model. In Second Meeting of the North American Chapter of the Association for Computational Linguistics. https://www.aclweb.org/anthology/N01-1021

[bib56] Hale, J. T. (2014). Automaton theories of human sentence comprehension. CSLI Publications, Center for the Study of Language and Information. Retrieved July 1, 2022, from https://csli.sites.stanford.edu/publications/csli-studies-computational-linguistics/automatontheories-human-sentence-comprehension.

[bib57] Hao, Y., Mendelsohn, S., Sterneck, R., Martinez, R., & Frank, R. (2020). Probabilistic predictions of people perusing: Evaluating metrics of language model performance for psycholinguistic modeling. In Proceedings of the Workshop on Cognitive Modeling and Computational Linguistics (pp. 75–86). 10.18653/v1/2020.cmcl-1.10

[bib58] Hochreiter, S., & Schmidhuber, J. (1997). Long short-term memory. Neural Computation, 9(8), 1735–1780. 10.1162/neco.1997.9.8.1735 9377276

[bib59] Hofmann, M. J., Biemann, C., & Remus, S. (2017). Benchmarking n-grams, topic models and recurrent neural networks by cloze completions, eegs and eye movements. In Cognitive Approach to Natural Language Processing (pp. 197–215). Elsevier. 10.1016/B978-1-78548-253-3.50010-X

[bib60] Hofmann, M. J., Remus, S., Biemann, C., Radach, R., & Kuchinke, L. (2022). Language models explain word reading times better than empirical predictability. Frontiers in Artificial Intelligence, 4, 730570. 10.3389/frai.2021.730570, 35187472 PMC8847793

[bib61] Hu, X., Mi, H., Li, L., & de Melo, G. (2022). Fast-R2D2: A pretrained recursive neural network based on pruned CKY for grammar induction and text representation. 10.48550/ARXIV.2203.00281

[bib62] Hu, X., Mi, H., Wen, Z., Wang, Y., Su, Y., Zheng, J., & de Melo, G. (2021). R2D2: Recursive transformer based on differentiable tree for interpretable hierarchical language modeling. In Proceedings of the 59th Annual Meeting of the Association for Computational Linguistics and the 11th International Joint Conference on Natural Language Processing (Volume 1: Long Papers) (pp. 4897–4908). 10.18653/v1/2021.acl-long.379

[bib63] Jaeger, T. F. (2006). Redundancy and syntactic reduction in spontaneous speech [Unpublished doctoral dissertation]. Stanford University.

[bib64] Jäger, L. A., Engelmann, F., & Vasishth, S. (2015). Retrieval interference in reflexive processing: Experimental evidence from Mandarin, and computational modeling. Frontiers in Psychology, 6, 617. 10.3389/fpsyg.2015.00617, 26074829 PMC4444751

[bib65] Jin, L., & Schuler, W. (2020). Memory-bounded neural incremental parsing for psycholinguistic prediction. In Proceedings of the 16th International Conference on Parsing Technologies and the IWPT 2020 Shared Task on Parsing into Enhanced Universal Dependencies (pp. 48–61). 10.18653/v1/2020.iwpt-1.6

[bib66] Jurafsky, D. (1996). A probabilistic model of lexical and syntactic access and disambiguation. Cognitive Science, 20(2), 137–194. 10.1207/s15516709cog2002_1

[bib67] Kuribayashi, T., Oseki, Y., Brassard, A., & Inui, K. (2022). Context limitations make neural language models more human-like. In Proceedings of the 2022 Conference on Empirical Methods in Natural Language Processing (pp. 10421–10436). Retrieved April 30, 2023, from https://aclanthology.org/2022.emnlp-main.712.

[bib68] Laverghetta, A., Nighojkar, A., Mirzakhalov, J., & Licato, J. (2022). Predicting human psychometric properties using computational language models. In M. Wiberg, D. Molenaar, J. González, J.-S. Kim, & H. Hwang (Eds.), Quantitative Psychology (pp. 151–169). Springer International Publishing. 10.1007/978-3-031-04572-1_12

[bib69] Levy, R. (2005). Probabilistic models of word order and syntactic discontinuity. https://www.proquest.com/dissertations-theses/probabilistic-models-word-ordersyntactic/docview/305432573/se-2?accountid=12339

[bib70] Levy, R. (2008a). Expectation-based syntactic comprehension. Cognition, 106(3), 1126–1177. 10.1016/j.cognition.2007.05.006, 17662975

[bib71] Levy, R. (2008b). A noisy-channel model of human sentence comprehension under uncertain input. In Proceedings of the 2008 Conference on Empirical Methods in Natural Language Processing (pp. 234–243). https://aclanthology.org/D08-1025

[bib72] Levy, R. (2011). Integrating surprisal and uncertain-input models in online sentence comprehension: Formal techniques and empirical results. In Proceedings of the 49th Annual Meeting of the Association for Computational Linguistics: Human Language Technologies (pp. 1055–1065). https://aclanthology.org/P11-1106

[bib73] Levy, R. (2013). Memory and surprisal in human sentence comprehension. In R. P. G. van Gompel (Ed.), Sentence processing (pp. 78–114). Psychology Press. https://www.mit.edu/%20rplevy/papers/levy-2013-memory-and-surprisal-corrected.pdf

[bib74] Levy, R. (2018). Communicative efficiency, uniform information density, and the rational speech act theory. In C. Kalish, J. Z. Martina Rau, & T. Rogers (Eds.), Proceedings of the 40th Annual Meeting of the Cognitive Science Society (pp. 684–689). https://cogsci.mindmodeling.org/2018/papers/0146/

[bib75] Levy, R., Fedorenko, E., & Gibson, E. (2013). The syntactic complexity of Russian relative clauses. Journal of Memory and Language, 69(4), 461–495. 10.1016/j.jml.2012.10.005, 24711687 PMC3975271

[bib76] Levy, R., & Jaeger, T. F. (2006). Speakers optimize information density through syntactic reduction. In B. Schölkopf, J. C. Platt, & T. Hofmann (Eds.), Proceedings of the Twentieth Annual Conference on Neural Information Processing Systems (pp. 849–856). Cambridge, MA: MIT Press. https://proceedings.neurips.cc/paper/2006/hash/c6a01432c8138d46ba39957a8250e027-Abstract.html

[bib77] Levy, R., Reali, F., & Griffiths, T. L. (2008). Modeling the effects of memory on human online sentence processing with particle filters. In D. Koller, D. Schuurmans, Y. Bengio, & L. Bottou (Eds.), Advances in neural information processing systems 21, Proceedings of the twenty-second annual conference on neural information processing systems, Vancouver, British Columbia, Canada, December 8–11, 2008 (pp. 937–944). Curran Associates, Inc. https://proceedings.neurips.cc/paper/2008/hash/a02ffd91ece5e7efeb46db8f10a74059-Abstract.html

[bib78] Lewis, R. L., & Vasishth, S. (2005). An activation-based model of sentence processing as skilled memory retrieval. Cognitive Science, 29(3), 375–419. 10.1207/s15516709cog0000_25, 21702779

[bib79] Lo, S., & Andrews, S. (2015). To transform or not to transform: Using generalized linear mixed models to analyse reaction time data. Frontiers in Psychology, 6, 1171. 10.3389/fpsyg.2015.01171, 26300841 PMC4528092

[bib80] Lowder, M. W., Choi, W., Ferreira, F., & Henderson, J. M. (2018). Lexical predictability during natural reading: Effects of surprisal and entropy reduction. Cognitive Science, 42(S4), 1166–1183. 10.1111/cogs.12597, 29442360 PMC5988918

[bib81] Luong, T., O’Donnell, T., & Goodman, N. (2015). Evaluating models of computation and storage in human sentence processing. In Proceedings of the Sixth Workshop on Cognitive Aspects of Computational Language Learning (pp. 14–21). 10.18653/v1/W15-2403

[bib82] Marcus, M. P. (1978). A theory of syntactic recognition for natural language. https://hdl.handle.net/1721.1/16176

[bib83] Marr, D. (1982). Vision: A computational investigation into the human representation and processing of visual information. W. H. Freeman.

[bib84] Marslen-Wilson, W. D. (1973). Linguistic structure and speech shadowing at very short latencies. Nature, 244(5417), 522–523. 10.1038/244522a0, 4621131

[bib85] Marslen-Wilson, W. D. (1975). Sentence perception as an interactive parallel process. Science, 189(4198), 226–228. 10.1126/science.189.4198.226, 17733889

[bib86] McDonald, S. A., & Shillcock, R. C. (2003a). Low-level predictive inference in reading: The influence of transitional probabilities on eye movements. Vision Research, 43(16), 1735–1751. 10.1016/s0042-6989(03)00237-2, 12818344

[bib87] McDonald, S. A., & Shillcock, R. C. (2003b). Eye movements reveal the on-line computation of lexical probabilities during reading. Psychological Science, 14(6), 648–652. 10.1046/j.0956-7976.2003.psci_1480.x, 14629701

[bib88] Meister, C., Cotterell, R., & Vieira, T. (2020). If beam search is the answer, what was the question? In Proceedings of the 2020 Conference on Empirical Methods in Natural Language Processing (EMNLP) (pp. 2173–2185). 10.18653/v1/2020.emnlp-main.170

[bib89] Meister, C., Pimentel, T., Haller, P., Jäger, L., Cotterell, R., & Levy, R. (2021). Revisiting the uniform information density hypothesis. In Proceedings of the 2021 Conference on Empirical Methods in Natural Language Processing. 10.18653/v1/2021.emnlp-main.74

[bib90] Meister, C., Vieira, T., & Cotterell, R. (2020). Best-first beam search. Transactions of the Association for Computational Linguistics, 8, 795–809. 10.1162/tacl_a_00346

[bib91] Merkx, D., & Frank, S. L. (2021). Human sentence processing: Recurrence or attention? In Proceedings of the Workshop on Cognitive Modeling and Computational Linguistics. 10.18653/v1/2021.cmcl-1.2

[bib92] Miller, G. A., & Chomsky, N. (1963). Finitary models of language users. In D. Luce (Ed.), Handbook of mathematical psychology (pp. 2–419). John Wiley & Sons. https://www.semanticscholar.org/paper/Finitary-models-of-language-users-Miller-Chomsky/4f3695d5dd36bb0abd91c02d2725463fca556f46

[bib93] Mitchell, D. C. (1984). An evaluation of subject-paced reading tasks and other methods for investigating immediate processes in reading 1. In New Methods in Reading Comprehension Research. Routledge.

[bib94] Mitchell, J., Lapata, M., Demberg, V., & Keller, F. (2010). Syntactic and semantic factors in processing difficulty: An integrated measure. In Proceedings of the 48th Annual Meeting of the Association for Computational Linguistics (pp. 196–206). https://www.aclweb.org/anthology/P10-1021

[bib95] Narayanan, S., & Jurafsky, D. (2001). A Bayesian model predicts human parse preference and reading times in sentence processing. In Advances in Neural Information Processing Systems (Vol. 14). Retrieved June 28, 2022, from https://proceedings.neurips.cc/paper/2001/hash/f15d337c70078947cfe1b5d6f0ed3f13-Abstract.html.

[bib96] Narayanan, S., & Jurafsky, D. (2004). A Bayesian model of human sentence processing [Unpublished manuscript]. https://web.stanford.edu/~jurafsky/narayananjurafsky04.pdf

[bib97] Newell, A., & Paul, R. (1981). Mechanisms of skill acquisition and the law of practice. In J. R. Anderson (Ed.), Cognitive Skills and Their Acquisition. Psychology Press. 10.4324/9780203728178-6

[bib98] Nicenboim, B., & Vasishth, S. (2018). Models of retrieval in sentence comprehension: A computational evaluation using Bayesian hierarchical modeling. Journal of Memory and Language, 99, 1–34. 10.1016/j.jml.2017.08.004

[bib99] Nivre, J. (2008). Algorithms for Deterministic Incremental Dependency Parsing. Computational Linguistics, 34(4), 513–553. 10.1162/coli.07-056-R1-07-027

[bib100] Oh, B.-D., Clark, C., & Schuler, W. (2022). Comparison of structural parsers and neural language models as surprisal estimators. Frontiers in Artificial Intelligence, 5, 777963. 10.3389/frai.2022.777963, 35310956 PMC8929193

[bib101] Oh, B.-D., & Schuler, W. (2023a). Why does surprisal from larger transformer-based language models provide a poorer fit to human reading times? Transactions of the Association for Computational Linguistics, 11, 336–350. 10.1162/tacl_a_00548

[bib102] Oh, B.-D., & Schuler, W. (2023b). Transformer-based LM surprisal predicts human reading times best with about two billion training tokens. arXiv: 2304.11389 [cs]. 10.48550/arXiv.2304.11389

[bib103] Piantadosi, S. T. (2014). Zipf’s word frequency law in natural language: A critical review and future directions. Psychonomic Bulletin & Review, 21(5), 1112–1130. 10.3758/s13423-014-0585-6, 24664880 PMC4176592

[bib104] R Core Team. (2021). R: A language and environment for statistical computing. Manual. R Foundation for Statistical Computing. Vienna, Austria. https://www.R-project.org/

[bib105] Radford, A., Narasimhan, K., Salimans, T., & Sutskever, I. (2018). Improving language understanding by generative pre-training. https://cdn.openai.com/research-covers/languageunsupervised/language_understanding_paper.pdf

[bib106] Radford, A., Wu, J., Child, R., Luan, D., Amodei, D., & Sutskever, I. (2019). Language models are unsupervised multitask learners. https://cdn.openai.com/better-languagemodels/language_models_are_unsupervised_multitask_learners.pdf

[bib107] Rasmussen, N. E., & Schuler, W. (2018). Left-corner parsing with distributed associative memory produces surprisal and locality effects. Cognitive Science, 42(S4), 1009–1042. 10.1111/cogs.12511, 28763111

[bib108] Reichle, E. D., Rayner, K., & Pollatsek, A. (2003). The E-Z Reader model of eye-movement control in reading: Comparisons to other models. Behavioral and Brain Sciences, 26(4), 445–476. 10.1017/s0140525x03000104, 15067951

[bib109] Rigby, R. A., & Stasinopoulos, D. M. (2005). Generalized additive models for location, scale and shape. Journal of the Royal Statistical Society: Series C (Applied Statistics), 54(3), 507–554. 10.1111/j.1467-9876.2005.00510.x

[bib110] Roark, B. (2001). Probabilistic top-down parsing and language modeling. Computational Linguistics, 27(2), 249–276. 10.1162/089120101750300526

[bib111] Roark, B., Bachrach, A., Cardenas, C., & Pallier, C. (2009). Deriving lexical and syntactic expectation-based measures for psycholinguistic modeling via incremental top-down parsing. In Proceedings of the 2009 Conference on Empirical Methods in Natural Language Processing (pp. 324–333). https://www.aclweb.org/anthology/D09-1034

[bib112] Rosenkrantz, D. J., & Lewis, P. M. (1970). Deterministic left corner parsing. In 11th Annual Symposium on Switching and Automata Theory (Swat 1970) (pp. 139–152). 10.1109/SWAT.1970.5

[bib113] Sanz-Alonso, D. (2018). Importance sampling and necessary sample size: An information theory approach. SIAM/ASA Journal on Uncertainty Quantification, 6(2), 867–879. 10.1137/16M1093549

[bib114] Shain, C., Meister, C., Pimentel, T., Cotterell, R., & Levy, R. P. (2022). Large-scale evidence for logarithmic effects of word predictability on reading time. 10.31234/osf.io/4hyna PMC10927576 38422017

[bib115] Shain, C., & Schuler, W. (2021). Continuous-time deconvolutional regression for psycholinguistic modeling. Cognition, 215, 104735. 10.1016/j.cognition.2021.104735, 34303182

[bib116] Shain, C., & Schuler, W. (2022). A deep learning approach to analyzing continuous-time systems. arXiv: 2209.12128 [cs, stat]. 10.48550/arXiv.2209.12128 PMC10962694 38528907

[bib117] Smith, N. J., & Levy, R. (2008a). Optimal processing times in reading: A formal model and empirical investigation. In Proceedings of the Annual Meeting of the Cognitive Science Society (Vol. 30, pp. 570–576). https://escholarship.org/uc/item/3mr8m3rf

[bib118] Smith, N. J., & Levy, R. (2008b). Probabilistic prediction and the continuity of language comprehension. In 9th Conference on Conceptual Structure, Discourse, and Language (CSDL9). https://papers.ssrn.com/sol3/papers.cfm?abstract_id=1295346

[bib119] Smith, N. J., & Levy, R. (2011). Cloze but no cigar: The complex relationship between cloze, corpus, and subjective probabilities in language processing. In Proceedings of the 33rd Annual Meeting of the Cognitive Science Society. https://escholarship.org/uc/item/69s3541f

[bib120] Smith, N. J., & Levy, R. (2013). The effect of word predictability on reading time is logarithmic. Cognition, 128(3), 302–319. 10.1016/j.cognition.2013.02.013, 23747651 PMC3709001

[bib121] Sóskuthy, M. (2021). Evaluating generalised additive mixed modelling strategies for dynamic speech analysis. Journal of Phonetics, 84, 101017. 10.1016/j.wocn.2020.101017

[bib122] Stabler, E. P. (2013). Two models of minimalist, incremental syntactic analysis. Topics in Cognitive Science, 5(3), 611–633. 10.1111/tops.12031, 23757195

[bib123] Staub, A. (2010). Eye movements and processing difficulty in object relative clauses. Cognition, 116(1), 71–86. 10.1016/j.cognition.2010.04.002, 20427040

[bib124] Stolcke, A. (1995). An efficient probabilistic context-free parsing algorithm that computes prefix probabilities. Computational Linguistics, 21(2), 165–201. https://www.aclweb.org/anthology/J95-2002

[bib125] Tanenhaus, M. K., Spivey-Knowlton, M. J., Eberhard, K. M., & Sedivy, J. C. (1995). Integration of visual and linguistic information in spoken language comprehension. Science, 268(5217), 1632–1634. 10.1126/science.7777863 7777863

[bib126] Taylor, W. L. (1953). ‘Cloze procedure’: A new tool for measuring readability. Journalism Quarterly, 30(4), 415–433. 10.1177/107769905303000401

[bib127] Traxler, M. J., Morris, R. K., & Seely, R. E. (2002). Processing subject and object relative clauses: Evidence from eye movements. Journal of Memory and Language, 47(1), 69–90. 10.1006/jmla.2001.2836

[bib128] Vani, P., Wilcox, E. G., & Levy, R. (2021). Using the interpolated maze task to assess incremental processing in English relative clauses. In Proceedings of the 43rd Annual Meeting of the Cognitive Science Society. Retrieved March 9, 2023, from https://escholarship.org/uc/item/3x34x7dz.

[bib129] van Schijndel, M., & Linzen, T. (2021). Single-stage prediction models do not explain the magnitude of syntactic disambiguation difficulty. Cognitive Science, 45(6), e12988. 10.1111/cogs.12988 34170031

[bib130] Vasishth, S. (2006). On the proper treatment of spillover in real-time reading studies: Consequences for psycholinguistic theories. In Proceedings of the International Conference on Linguistic Evidence (pp. 96–100).

[bib131] Vasishth, S., & Engelmann, F. (2021). Sentence comprehension as a cognitive process: A computational approach (1st ed.). Cambridge University Press. 10.1017/9781316459560

[bib132] Vasishth, S., Nicenboim, B., Engelmann, F., & Burchert, F. (2019). Computational models of retrieval processes in sentence processing. Trends in Cognitive Sciences, 23(11), 968–982. 10.1016/j.tics.2019.09.003 31668586

[bib133] Vaswani, A., Shazeer, N., Parmar, N., Uszkoreit, J., Jones, L., Gomez, A. N., Kaiser, L., & Polosukhin, I. (2017). Attention is all you need. In I. Guyon, U. von Luxburg, S. Bengio, H. M. Wallach, R. Fergus, S. V. N. Vishwanathan, & R. Garnett (Eds.), Advances in neural information processing systems 30: Annual conference on neural information processing systems 2017, December 4–9, 2017, Long Beach, CA, USA (pp. 5998–6008). https://proceedings.neurips.cc/paper/2017/hash/3f5ee243547dee91fbd053c1c4a845aa-Abstract.html

[bib134] Vieira, T., & Eisner, J. (2017). Learning to prune: Exploring the frontier of fast and accurate parsing. Transactions of the Association for Computational Linguistics, 5, 263–278. 10.1162/tacl_a_00060

[bib135] Wang, B., & Komatsuzaki, A. (2021). GPT-J-6B: A 6 billion parameter autoregressive language model. https://github.com/kingoflolz/mesh-transformer-jax

[bib136] Wieling, M., Tomaschek, F., Arnold, D., Tiede, M., Bröker, F., Thiele, S., Wood, S. N., & Baayen, R. H. (2016). Investigating dialectal differences using articulography. Journal of Phonetics, 59, 122–143. 10.1016/j.wocn.2016.09.004

[bib137] Wilcox, E. G., Gauthier, J., Hu, J., Qian, P., & Levy, R. (2020). On the predictive power of neural language models for human real-time comprehension behavior. In Proceedings of the 42nd Annual Meeting of the Cognitive Science Society (pp. 1707–1713). https://www.cognitivesciencesociety.org/cogsci20/papers/0375/

[bib138] Wolf, T., Debut, L., Sanh, V., Chaumond, J., Delangue, C., Moi, A., Cistac, P., Rault, T., Louf, R., Funtowicz, M., Davison, J., Shleifer, S., von Platen, P., Ma, C., Jernite, Y., Plu, J., Xu, C., Le Scao, T., Gugger, S., … Rush, A. (2020). Transformers: State-of-the-art natural language processing. In Proceedings of the 2020 Conference on Empirical Methods in Natural Language Processing: System Demonstrations (pp. 38–45). 10.18653/v1/2020.emnlp-demos.6

[bib139] Wood, S. N. (2003). Thin plate regression splines. Journal of the Royal Statistical Society: Series B (Statistical Methodology), 65(1), 95–114. 10.1111/1467-9868.00374

[bib140] Wood, S. N. (2004). Stable and efficient multiple smoothing parameter estimation for generalized additive models. Journal of the American Statistical Association, 99(467), 673–686. 10.1198/016214504000000980

[bib141] Wood, S. N. (2011). Fast stable restricted maximum likelihood and marginal likelihood estimation of semiparametric generalized linear models. Journal of the Royal Statistical Society: Series B (Statistical Methodology), 73(1), 3–36. 10.1111/j.1467-9868.2010.00749.x

[bib142] Wood, S. N. (2017). Generalized additive models. Chapman and Hall/CRC. 10.1201/9781315370279

[bib143] Wood, S. N., Pya, N., & Säfken, B. (2016). Smoothing parameter and model selection for general smooth models. Journal of the American Statistical Association, 111(516), 1548–1563. 10.1080/01621459.2016.1180986

[bib144] Yang, K., & Deng, J. (2020). Strongly incremental constituency parsing with graph neural networks. In H. Larochelle, M. Ranzato, R. Hadsell, M. F. Balcan, & H. Lin (Eds.), Advances in neural information processing systems (Vol. 33, pp. 21687–21698). Curran Associates, Inc. https://proceedings.neurips.cc/paper/2020/file/f7177163c833dff4b38fc8d2872f1ec6-Paper.pdf

[bib145] Yngve, V. H. (1960). A model and an hypothesis for language structure. Proceedings of the American Philosophical Society, 104(5), 444–466. Retrieved March 10, 2023, from https://www.jstor.org/stable/985230.

[bib146] Zhang, S., Roller, S., Goyal, N., Artetxe, M., Chen, M., Chen, S., Dewan, C., Diab, M., Li, X., Lin, X. V., Mihaylov, T., Ott, M., Shleifer, S., Shuster, K., Simig, D., Koura, P. S., Sridhar, A., Wang, T., & Zettlemoyer, L. (2022). OPT: Open Pre-trained Transformer language models. arXiv:2205.01068 [cs]. 10.48550/arXiv.2205.01068

[bib147] Zhang, Y., & Clark, S. (2008). A tale of two parsers: Investigating and combining graph-based and transition-based dependency parsing. In Proceedings of the 2008 Conference on Empirical Methods in Natural Language Processing (pp. 562–571). https://aclanthology.org/D08-1059

